# Nanofibrous Scaffolds’ Ability to Induce Mesenchymal Stem Cell Differentiation for Soft Tissue Regenerative Applications

**DOI:** 10.3390/ph18020239

**Published:** 2025-02-11

**Authors:** Silvia Pisani, Aleksandra Evangelista, Luca Chesi, Stefania Croce, Maria Antonietta Avanzini, Rossella Dorati, Ida Genta, Marco Benazzo, Patrizia Comoli, Bice Conti

**Affiliations:** 1 Department of Drug Sciences, University of Pavia, 27100 Pavia, Italy; chesi.luca@gmail.com (L.C.); rossella.dorati@unipv.it (R.D.); ida.genta@unipv.it (I.G.); bice.conti@unipv.it (B.C.); 2 Otorhinolaryngology Unit, Department of Surgical Sciences, Fondazione IRCCS Policlinico San Matteo, 27100 Pavia, Italy; a.evangelista@smatteo.pv.it (A.E.); m.benazzo@smatteo.pv.it (M.B.); 3 Department of Clinical, Surgical, Diagnostic & Pediatric Sciences, Fondazione IRCCS Policlinico San Matteo, 27100 Pavia, Italy; s.croce@smatteo.pv.it (S.C.); ma.avanzini@smatteo.pv.it (M.A.A.); p.comoli@smatteo.pv.it (P.C.)

**Keywords:** mesenchymal stem cells, electrospinning, nanofibrous scaffolds, differentiation

## Abstract

Mesenchymal stem cells (MSCs) have gained recognition as a highly versatile and promising cell source for repopulating bioengineered scaffolds due to their inherent capacity to differentiate into multiple cell types. However, MSC implantation techniques have often yielded inconsistent clinical results, underscoring the need for advanced approaches to enhance their therapeutic efficacy. Recent developments in three-dimensional (3D) bioengineered scaffolds have provided a significant breakthrough by closely mimicking the in vivo environment, addressing the limitations of traditional two-dimensional (2D) cell cultures. Among these, nanofibrous scaffolds have proven particularly effective, offering an optimal 3D framework, growth-permissive substrates, and the delivery of trophic factors crucial for MSC survival and regeneration. Furthermore, the selection of appropriate biomaterials can amplify the paracrine effects of MSCs, promoting both proliferation and targeted differentiation. The synergistic combination of MSCs with nanofibrous scaffolds has demonstrated remarkable potential in achieving repair, regeneration, and tissue-specific differentiation with enhanced safety and efficacy, paving the way for routine clinical applications. In this review, we examine the most recent studies (2013–2023) that explore the combined use of MSCs and nanofibrous scaffolds for differentiation into cardiogenic, epithelial, myogenic, tendon, and vascular cell lineages. Using PubMed, we identified and analyzed 275 relevant articles based on the search terms “Nanofibers”, “Electrospinning”, “Mesenchymal stem cells”, and “Differentiation”. This review highlights the critical advancements in the use of nanofibrous scaffolds as a platform for MSC differentiation and tissue regeneration. By summarizing key findings from the last decade, it provides valuable insights for researchers and clinicians aiming to optimize scaffold design, MSC integration, and translational applications. These insights could significantly influence future research directions and the development of more effective regenerative therapies.

## 1. Introduction

Tissue engineering (TE) integrates knowledge from cell biology and material science to replicate the physical and chemical characteristics of native tissue, aiming to restore its function after injury [1].

Mesenchymal stem cells (MSCs) are multipotent progenitor cells found in the skin, dental pulp, adipose tissue, bone marrow, and umbilical cord. These cells can be isolated and expanded in vitro. According to the criteria defined by the International Society of Cell Therapy’s Committee on Mesenchymal and Tissue Stem Cells, they must be adherent to plastic and exhibit the typical spindle shape morphology, and they must express CD105, CD73, and CD90 cell surface markers and lack the expression of CD45, CD34, CD14, CD11b, CD79a, CD19, and HLA Class II. Moreover, they must maintain the ability to differentiate into osteoblasts, adipocytes, and chondrocytes [2,3].

Recent findings have highlighted how physical and mechanical stimuli can influence cell fate, accelerating the understanding of regulatory mechanisms that govern stem cell differentiation. Advances in scaffold micro-/nanofabrication have enabled the development of advanced materials that mimic the biophysical characteristics of the body’s environment. Since the cellular microenvironment directly impacts cell structure and function, material properties, such as stiffness, topology, and surface chemistry, have been studied to modulate the signaling pathways involved in stem cell differentiation [4,5].

The fabrication of three-dimensional (3D) porous scaffolds produces structures capable of supporting cell survival, guiding cell proliferation, promoting functional differentiation, and facilitating the targeting of transplanted replacement or supporting cells. Scaffolds, due to their architecture, offer proper cell positioning and adhesion, as well as the deposition of the Extracellular Matrix (ECM) [6]. Scaffolds also ensure the efficient transport of gases, essential nutrients, and regulatory factors to support cell proliferation, survival, and differentiation [7]. The porosity of three-dimensional scaffolds must be optimized based on the specific cell type used and its intended function [8]. An example reported that cell infiltration for dermal fibroblasts is enhanced if scaffolds with 34.4% porosity and an average pore size of 11 μm are used; the proliferation of human MSCs is promoted by scaffolds with a porosity of 88% and a pore size of 500 μm [9,10].

Scaffolds can be categorized based on their material origin into natural (e.g., collagen, chitosan, glycosaminoglycans, hyaluronic acid, decellularized matrix), synthetic (e.g., bioceramics, metals, polyesters), and composite categories, which are made by combining materials from different origins [11]. It is essential that all materials used must be biocompatible (biomaterials) to avoid systemic toxicity (cytotoxicity, genotoxicity, mutagenicity, carcinogenicity and immunogenicity) once implanted [12]. Furthermore, bio-scaffolds should possess adjustable mechanical strength, a large surface area, and surface characteristics that replicate the physical and chemical properties of the ECM to support cell adhesion, proliferation, and differentiation [13,14]. Concerning differentiation, scaffolds play a fundamental role in driving MSC differentiation in specific cell lines. Among all scaffold manufacturing techniques, electrospinning is widely used due to its ability to produce nanofibers, mimicking the natural sub-micron features of the native tissue. The ultrafine fibers and their alignment create an extracellular matrix-like environment that supports the regulation of cellular functions and guides the differentiation fate of MSCs [15]. Studies have shown that nanofibers produced from different polymer precursors, along with their resulting scaffold architectures, compositions, and properties, significantly influence MSC activities by enhancing cell–cell and cell–material interactions [16,17].

In this review, we analyzed various polymers’ nanofibrous scaffolds, and their abilities to induce MSC differentiation were analyzed. Information on scaffolds’ materials and mechanical properties was identified to correlate their ability to induce MSC differentiation in cardiogenic, epithelial, myogenic, tendon, and vascular lines.

**Figure 1:**
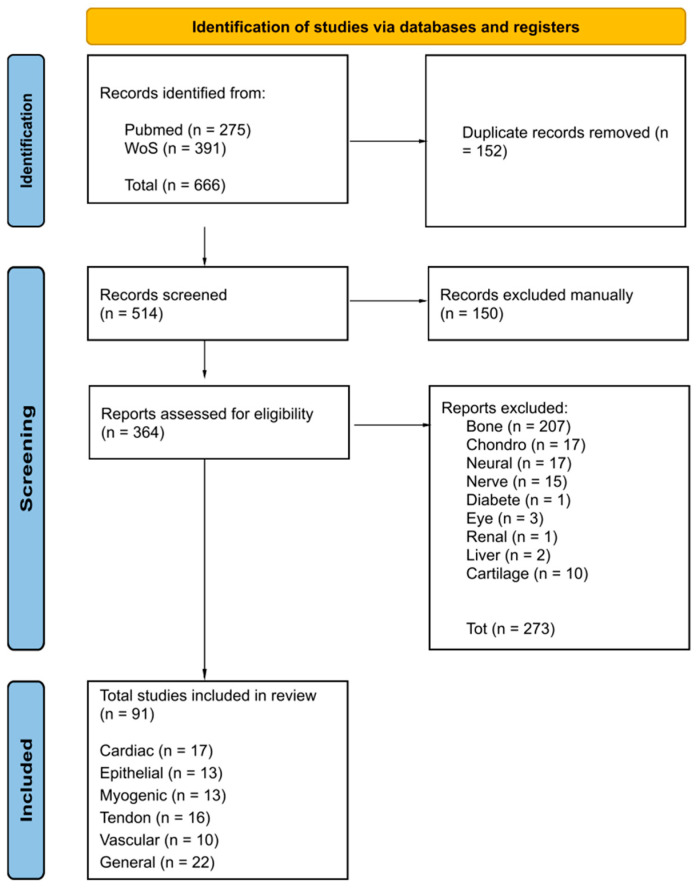
A systematic search conducted using PRISMA 2020 flow diagram for updated systematic reviews, which included searches of databases and registers only. The search query (Nanofibers) and (Electrospinning) and (Mesenchymal stem cells) and (Differentiation) was used on PubMed and Web of Science over the last 10 years (2014–2024). A total number of 666 articles found in the last 10 years (2014–2024) were registered. After a systematic process of applying exclusion and inclusion criteria, 91 studies were included in this review.

A systematic search was conducted using the PRISMA 2020 protocol and the following search query: (Nanofibers) and (Electrospinning) and (Mesenchymal stem cells) and (Differentiation) on PubMed and Web of Science (WoS). The inclusion criteria included full-text articles that used electrospun nanofibers for MSC differentiation in cardiogenic, epithelial, myogenic, tendon, and vascular lines. Studies not specifically addressing the use of electrospun nanofibers with the aim of MSC differentiation were excluded, as were full-text articles that used electrospun nanofibers for MSC differentiation in lines not indicated above (e.g., bone, neural, nerve, chondrogenic, and other applications). A total of 666 papers were found in the search conducted over the last 10 years (2014–2024) (Figure 1). A total of 152 duplicate records were removed. A total of 514 records were screened, and another 150 were excluded by a human reviewer because they did not satisfy the criteria cited above. Of the 364 remaining items assessed for eligibility, 273 were excluded because they referred to the differentiation of MSCs into regenerative lineages not of interest for this review. Among the remaining 91 full-text articles considered and cited for the review, 22 papers focused on general concepts, 17 referred to cardiac differentiation, 13 referred to epithelial differentiation, 13 referred to myogenic differentiation, 16 referred to tendon differentiation, and 10 referred to vascular tissues.

## 2. Nanofibrous Scaffolds Inducing MSC Differentiation

### 2.1. Cardiogenic Differentiation

Cardiovascular diseases are the primary cause of death and disability globally. In recent years, therapies based on stem cells have demonstrated significant potential in the treatment of ischemic heart conditions. Additionally, the differentiation of stem cells into cardiomyocytes (CMs) offers a viable solution to address the challenge of limited cell sources. Among the emerging therapeutic approaches, myocardial tissue engineering stands out as a highly promising strategy for restoring cardiac function following extensive heart damage. The success of this approach heavily relies on the synergistic integration of biomaterials, cellular components, and scaffold design in the creation of engineered heart tissue [18].

**Figure 2:**
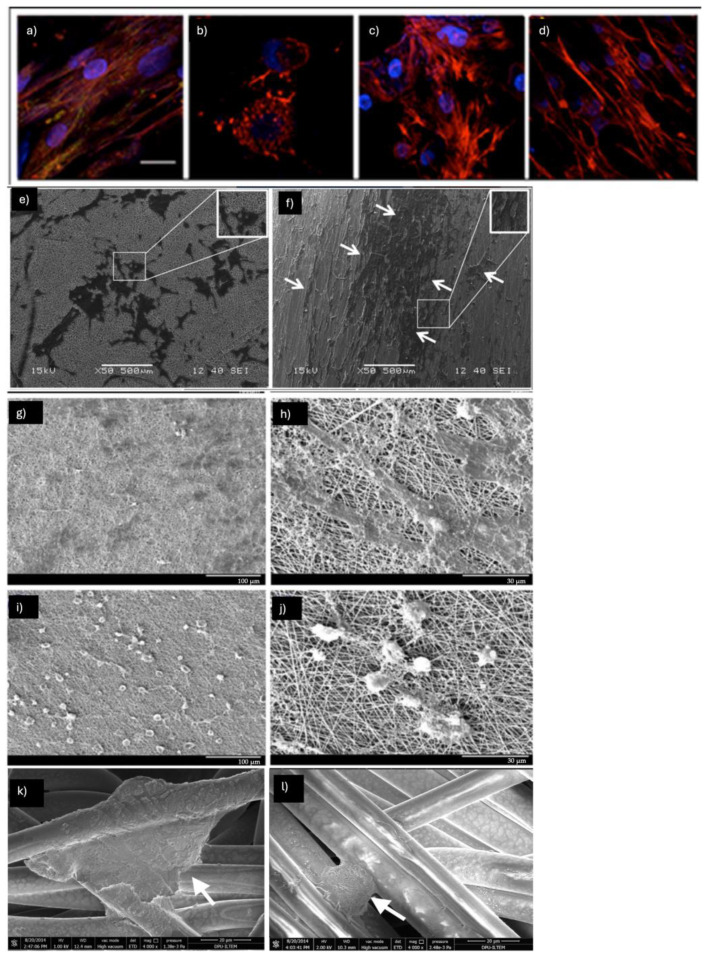
(**a**–**d**) Immunofluorescent staining of differentiated hMSCs for paxillin (green), F-actin (red), and nucleus (blue) in the presence of cytoskeletal inhibitors on PCL-A nanofibers. (**a**) Control; (**b**) Cells treated with F-actin inhibitor, cytochalasin D; (**c**) Cells treated with nocodazole; (**d**) Cells treated with Y-27632, a ROCK inhibitor. In panels (**c**,**d**), the cells adopt a tetragonal morphology and show a decrease in paxillin expression. Images obtained by [19]. (**e**) Random orientation of cells on a nonaligned nanofiber; (**f**) Well-oriented stem cells morphology on an aligned nanofiber matrix at day 4. Arrow indicates the MSCs aligned along fibers. Images obtained by [20]. (**g**–**j**) SEM micrographs of MSCs after differentiation to cardiomyocytes in PVA-CS-CNT3 (**g**,**h**) and PVA-CS-CNT5 (**i**,**j**) scaffolds after 14 days of exposure to electrical stimulation and the differentiation medium. Images obtained by [23]. (**k**,**l**) PU electrospun nanofibers and cardiomyocyte-like cells on (**k**) NGF (–) and (**l**) NGF (+) PU. Images obtained by [34].

The choice of support to direct cell differentiation plays an important role. In a study conducted by L.D. Ghosh et al., the molecular events underlying topography-mediated cardiomyogenesis of stem cells on PCL (15% *w*/*v*) nanofibrous scaffolds compared to PCL films (Figure 2a–d) [19]. The results showed that human MSCs cultured on random nanofibers exhibited increased expression of key cardiac markers, including cardiac actinin, cardiac troponin, and β-myocardial heavy chain, compared to those seeded on 2D films. Moreover, enhanced differentiation was highlighted for cells on aligned nanofibrous (657 ± 151 nm) scaffolds compared to the random (774 ± 80 nm) ones, showing that cells on the aligned fibers assumed a morphology that is typical of cardiomyocytes in vivo. Interestingly, the enhanced differentiation observed on aligned nanofibers was linked to elevated levels of the histone deacetylase SIRT6 and reduced levels of acetylated histone H3K9, suggesting the involvement of epigenetic regulation. The influence of fiber orientation was further investigated. Random (840  ±  23 nm) and aligned (512  ±  88 nm) PCL nanofibrous matrices implemented with 5-aza (5 μM) were tested with MSC and human-induced pluripotent stem cells (hiPSCs) for their ability to induce cardiomyocyte differentiation. Cells cultured on aligned fibers showed anisotropic behavior and, upon differentiation, expressed cardiac-specific cTnT and α-actinin proteins and showed higher synchronized beating (Figure 2e,f) [20].

Tambrchi et al. developed electrospun PLA-PCL (75:25, *w*/*w*) bioactive scaffolds for the cardiomyocyte differentiation of adipose-derived human Ad-MSCs (hAd-MSCs). In their study, they also evaluated the effect of 5-aza and transforming growth factor-β (TGF-β) on scaffold implementation [21]. Efficient cardiomyocyte differentiation was achieved after 21 days of incubation on supplemented PLA-PCL scaffolds. Real-time PCR for cardiac-specific genes, including cardiac troponin I (cTnI), GATA4, MYH7, and NKX2.5, was performed to confirm the differentiation.

Poly-caprolactone (PCL)/polyaniline (PANI) nano fibrous scaffolds prepared by electrospinning and human adipose-derived mesenchymal stem cells (Ad-MSCs) were used to study cardiomyocyte differentiation [22]. The results from the MTT assay and SEM analysis on Ad-MSC cultures demonstrated that the cells successfully proliferated on the PCL/PANI scaffolds, confirming the biocompatibility of the nanofibers and their ability to support cell growth and adhesion. After 21 days of induced cardiomyocyte differentiation using both agents, real-time PCR analysis showed an increase in the expression levels of the Gata4, troponin I, MYH-7, and NKX2.5 genes in cells cultured on the PCL/PANI scaffolds. Additionally, flow cytometry confirmed the expression of troponin I. Mobini et al. developed a conductive nanofibrous scaffold made of polyvinyl alcohol (PVA), chitosan (Ch), and varying concentrations of carbon nanotubes (CNT) for cardiovascular tissue engineering (Figure 2g–j) [23]. They found that 1% of CNT imparts optimal properties for the cardiac differentiation of rat mesenchymal stem cells (MSCs). Cardiac markers (Nkx2.5, troponin I, and β-MHC) detected by real-time PCR, showed a significant increase (>3-fold) in comparison to the control group (PVA-CS scaffolds).

Gold nanoparticles (AuNPs- 25 ± 5 nm) were embedded in polycaprolactone (PCL), vitamin B_12_ (VB_12_), aloe vera (AV) and silk fibroin (SF) nanofibrous scaffolds to enhance MSC differentiation into the cardiac lineage [24]. A final concentration of PCL/SF/AV/Vit B12/GNP (10, 2.5, 2.5, 0.5, 0.5%) was used to prepare electrospun scaffolds for further co-culturing cardiomyocytes and mesenchymal stem cells. Mechanical properties were also evaluated (2.56 MPa) to demonstrate that the composition of the scaffold and the microenvironment play crucial roles in guiding MSCs toward a cardiac lineage.

Gold was also used by another group to produce hybrid conductive electrospun scaffolds composed of bovine serum albumin/polyvinyl alcohol (BSA/PVA/AuNps) [25]. MSCs differentiated on BSA/PVA/Au (2:1:0.1) nanofibers displayed the characteristic multinucleated morphology and expressed cardiac proteins such as actinin, cTnT, and Cx43, indicating that the AuNPs-loaded nanofibrous scaffolds promote differentiation and enhance cell-to-cell communication through gap junctions, thereby supporting cardiac tissue regeneration.

Collagen is the primary structural protein in the myocardium, contributing to tissue strength and integrity, as well as influencing cellular orientation and cell–cell and cell–matrix interactions. For these reasons, core-shell electrospun nanofibers were produced using poly (glycerol sebacate)/collagen (PGS/collagen) to be tested in a co-culture system with cardiac cells and MSCs. Type I collagen was used to obtain randomly oriented electrospun nanofibers (789 ± 162 nm) to promote cardiomyogenic differentiation of human bone marrow-derived mesenchymal stem cell (BM-MSC) spheroids [26]. Cardiomyogenic markers (GATA4, cardiac troponin I, and myosin heavy chain (MHC)) were identified. Moreover, an interesting highlight of this study relates to the significant cellular alignment, irrespective of the underlying random orientation of the nanofibers.

Three-dimensional-aligned polycaprolactone scaffolds coated with different ECM proteins (human collagen, human fibronectin, and basement membrane Matrigel) were used by Ghosh et al. to further promote the cardiomyogenesis of human MSCs. Among the various coatings tested, collagen-coated fibers were the most effective in promoting cardiomyogenesis, as evidenced by the increased expression of cardiac biomarkers, intracellular calcium flux, and sirtuin 6 (SIRT6) [27].

Gelatin was also combined with PCL and vascular endothelial growth factor (VEGF) to produce electrospun scaffolds (PCL/G-VEGF) that were able to induce human MSCs (hMSCs) to differentiate into cardiomyogenic cell lineages [28]. Immunofluorescence staining performed after 15 days revealed that hMSCs differentiated into cardiomyogenic cells on PCL-gelatin nanofibers, expressing higher levels of cardiac-specific proteins (α-actinin and cTnT) compared to MSCs differentiated on tissue culture plates (2D control). core-shell poly(glycerol sebacate)/fibrinogen/VEGF (PGS/Fbg/VEGF) scaffolds cellularized with MSCs were evaluated in an in vivo porcine model (infarcted myocardium) using echocardiography, histology, and immunohistochemistry [29]. After transplantation, MSCs on PGS/Fbg/VEGF expressed cardiac marker proteins, including troponin and actinin, as well as the endothelial cell marker protein CD31, indicating the differentiation of MSCs into both cardiac and endothelial cells. Furthermore, the scaffolds provided structural support to the infarcted left ventricle wall. Adipose tissue-derived mesenchymal stem cells (Ad-MSCs) seeded onto polyamide electrospun nanofibers (280 nm) were tested for their ability to reconstruct damaged cardiac tissue in isoprenaline-induced myocardial infarction (MI) in rats [30]. Electrocardiogram (ECG), biochemical analysis, molecular genetic analysis, and histological examination were performed to determine the therapeutic potential. A reduction in lactate dehydrogenase (LDH), creatine kinase-MB (CK-MB) enzyme activities, troponin T (cTnT), and connexin 43 (Cx43) levels was detected in rats treated with cellularized electrospun scaffolds compared to those treated with Ad-MSC alone.

Umbilical cord blood-derived MSCs (UCB-MSCs) were seeded on fibronectin (FN) functionalized PCL-aligned electrospun scaffolds. An echocardiogram performed on an MI rat model showed that, after 4 weeks, the UCB-MSCs seeded on FN-PCL nanofibers increased LV ejection fraction and fraction shortening compared to the non-treated control and acellular FN-PCL nanofiber samples [31].

The impact of the protein’s secondary structures in the substrates on the morphological transformation and differentiation of human bone marrow-derived mesenchymal stem cells (hBM-MSCs) was assessed. Silk fibroin-poly(ε-caprolactone) (SF-PCL) cardiac patches grafted with silk fibroin (SF) containing varying β-sheet contents (ranging from 20% to 44%) provided different levels of scaffold stiffness [32]. After 3 days in culture, hBM-MSCs migrated and underwent morphological transformation into 3D microtissues with diameters of approximately 150–200 μm on low-stiffness SP20 and SP30 patches (120 MPa), compared to high-stiffness patches (>160 MPa). These microtissues exhibited more extensive in vitro cardiomyogenesis than the 2D cell monolayer. An alternative strategy was performed by Wu et al., who designed polyacrylonitrile (PAN)-based hydrogel yarns composed of uniaxially aligned nanofibers and characterized by an anisotropic architecture and flexible and robust mechanical properties. This pH-stimulus responsive nanofiber-structured hydrogel yarns could be assembled into defined scaffold structures using subsequent processes [33]. Human adipose-derived MSCs (hAd-MSCs) cultured on hydrogel yarns exhibited a significantly higher expression of smooth muscle cell (SMC) differentiation-related genes when cultured in smooth muscle cell differentiation medium (SMC-DM) for 14 days. Hydrogel yarns seeded with embryonic chicken cardiomyocytes enhanced sarcomere organization and mimicked the cardiomyocyte bundles found in native myocardium, supporting the spontaneous pumping behavior of the cardiomyocytes.

Saltik et al., developed BM-MSC cellularized cardiac patches made using biocompatible nerve growth factor (NGF)-embedded polyurethane (PU) nanofiber (Figure 2k,l) [34]. Cardiomyogenic differentiation was induced by treating BM MSCs with 5-azacytidine for 24 h. Cells exhibiting a morphology resembling cardiomyocytes began to appear between days 14 and 21. By day 28, the differentiation into cardiomyocytes was confirmed through staining for cardiac troponin T.

Another cardiac patch fabricated by Shoba et al. was developed using a dual bioactive embedded nanofibrous patch via a coaxial electrospinning technique to mimic the topographical and chemical cues of the natural cardiac tissue [35]. The bioactive loaded core-shell nanofibers (600 ± 34 nm) were prepared by dissolving 100 μM of salvianolic acid B, per mL of polymer solution in PCL (core) and 2 mg of magnesium l-ascorbic acid 2 phosphate, salts in gelatin (shell) solution. The fabricated scaffold (Sal B-MAAP PCL/GEL) showed good adherence of cells with better morphology, increased proliferation and differentiation. Moreover, the dual structure provided myotube-inductive cues in a slow and sustained way to the cell surface receptors, leading to the increased expression of cardiac markers (α actinin, troponin T, and connexin-43).

Table 1 shows examples of cardiomyogenic differentiation induced on nanofibrous scaffolds specifying the type of nanofibers, the mechanical properties, the source of MSCs and the additional factors used to induce the differentiation.

### 2.2. Epithelial Differentiation

The skin, the largest organ of the human body, acts as a vital barrier against harmful substances and pathogens, while also playing a key role in the administration of therapeutic agents, such as dermal and transdermal medications. When the skin is damaged due to burns, injuries, chronic wounds, or diseases, its protective role is compromised, which can lead to serious outcomes, including disability, or even death. Therefore, progress in the field of skin tissue engineering is essential to tackle these challenges effectively [36].

**Figure 3:**
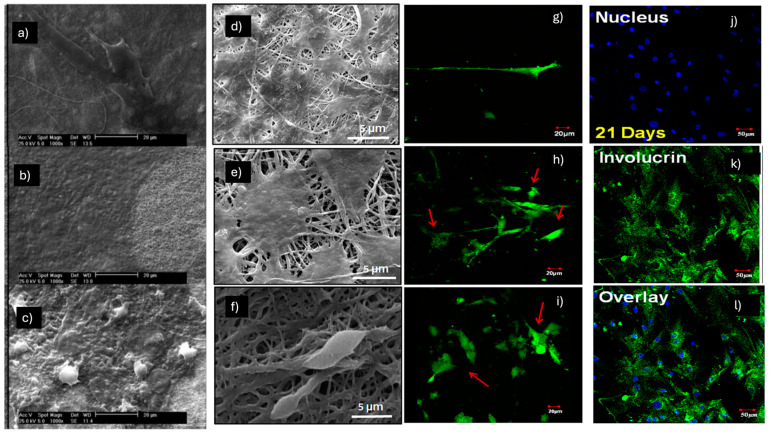
Morphology of (**a**) H-keratino/MSC on day 1; (**b**) day 14; and (**c**) day 21 on nylon and N-B.v NFM. Images obtained by [37]. (**d**–**f**) SEM photograph of MSC-derived keratinocyte attachment on fiber mats after 84 h of seeding. Images obtained by [38]. Live/dead imaging of BM-MSCs cultured in induction media at various time points: (**g**) 3 days; (**h**) 7 days; and (**i**) 14 days of culture. Red arrows highlight the morphological changes in BM-MSCs upon induction with epidermal differentiation media. (**j**–**l**) Immunostaining of CD105 and involucrin in epidermally differentiated BM-MSCs on PHBV nanofibers. Images obtained by [40].

Hosseinzadeh et al. observed a synergistic effect in promoting epithelial differentiation, starting from the co-culture of MSC-human keratinocytes (H-keratin) (at a 70:30 ratio) of a composite electrospun nylon66 nanofibrous scaffold loaded with Beta vulgaris (B. vulgaris), an extract of beet roots used in Iranian folk medicine to promote wound healing (Figure 3a–c) [37]. The analyses demonstrated that after 14 days of the co-culturing process, the gene expression of some epithelial markers, such as cytokeratin 10, cytokeratin 14 and involucrine, was increased using both the non-composite nylon66 nanofibrous scaffold (N NFS) and the composite electrospun nylon66 nanofibrous scaffold (N-B.v NFS) compared to the mono-cell culturing of h-keratinocyte used as the control. Furthermore, immunocytochemistry analysis revealed that 21 days after cell-seeding, hMSCs in co-culture systems expressed several epithelial markers like cytokeratin 14 and lorocrin strongly in the beet extract-loaded scaffold (N-B.v NFM) compared to the others. In conclusion, the study showed how a B.vulgaris-loaded electrospun nanofibrous composite scaffold and keratinocyte MSC co-culturing could synergistically stimulate the differentiation of MSCs toward epithelial lineage.

The wound healing potential for skin tissue regeneration of a co-electrospun hybrid of polyvinyl alcohol (PVA), chitosan (Ch), and silk fibers (Ch-PVA + Silk), seeded with bone marrow mesenchymal stem cell (BM-MSC)-derived keratinocytes, was analyzed both in vitro and in vivo in the study by Fathi et al. (Figure 3d–f) [38]. SEM analysis demonstrated that the Ch-PVA + silk fibrous mat was bead-free and smooth, without any branching, and the average diameter of the nanofibers was 1200 ± 321 nm. Before seeding MSC-derived keratinocytes on the fibrous scaffold surface, the differentiation induction of MSCs to keratinocytes involved the use of a specific medium containing 2 mM calcium chloride (CC), 5 μg/mL insulin, and 10 ng/mL recombinant human epidermal growth factor (EGF) and keratinocyte growth factor (KGF), which was changed every 3 days for up to 18 days. An evaluation of differentiation using phase contrast microscopy demonstrated a conformational change toward a polygonal and cobblestone morphology in the treated cells with differentiating media, similar to mice foreskin-derived keratinocytes used as a positive control. Furthermore, the results obtained via immunohistochemical staining demonstrated the expression positivity of the MSC-derived keratinocytes for epithelial protein markers, such as cytokeratin-19 (CK-19), involucrin (IVL), and vimentin (Vim), with results comparable to those of mice foreskin-derived keratinocytes. The Ch-PVA + silk fibrous mat was utilized for in vivo wound treatment experiments, showing that the scaffolds in the presence of MSC-derived keratinocytes could stimulate wound healing and skin tissue regeneration. For example, a considerable reduction in inflammatory cells and good collagen synthesis, which are fundamental in the wound healing process, were observed.

A chitin nanofiber (CNF) hydrogel was utilized in the study by Shou et al. to investigate its potential for inducing the differentiation of BM-MSCs to enhance cutaneous wound regeneration [39]. External inducers are traditionally used to drive the differentiation of BM-MSCs into desired cell types and promote wound healing. Conversely, the aim of this study was to explore a combinatorial strategy using BM-MSCs and CNF hydrogel without the need for soluble differentiation factors. CNF hydrogel, due to its physical properties and similarity to soft tissues like dermis and granulation tissue, was considered a suitable material. The results of the study showed that BM-MSCs cultured in CNF hydrogels exhibited a decrease in stemness-related gene expression, indicating their differentiation. This finding led to the hypothesis that N-acetylglucosamine, a major component of CNF hydrogel, may play a role in driving the differentiation of BM-MSCs instead of maintaining them in an undifferentiated state. Furthermore, wounds treated with cell-encapsulated CNF hydrogel showed accelerated healing compared to other groups used as controls at all time points.

In the study by Sundaramurthi et al., electrospun poly 3-hydroxybutyrate-co-3-hydroxyvalerate (PHBV) nanofiber scaffolds were assessed for their ability to support BM-MSC adhesion, proliferation, and epidermal differentiation (Figure 3g–l). This assessment was conducted in the presence of an epidermal induction medium containing 15% FBS, 1% P/S, 400 ng/mL hydrocortisone, 500 ng/mL insulin, 1 nM 3,3′,5-triiodo-L-thyronine (T3), 10 ng/mL EGF, 1 μM vitamin D3 (VD3), and 50 μg/mL L-AA [40]. Using scanning electron microscopy (SEM), BM-MSCs were found to adhere well to the PHBV nanofibers, and analysis via confocal microscopy of a live/dead staining confirmed their viability over time. Furthermore, the change in BM-MSC morphology from spindle-shaped to polygonal on PHBV nanofibers after 7 and 14 days of culture indicates their epidermal differentiation; this change may likely be attributed to the scaffold’s nano features and the presence of epidermal induction factors in the media. Another indication of successful differentiation is the upregulated expression, analyzed using RT-PCR, of the genes keratin 10 (early), filaggrin (intermediate), and involucrin (late), which are involved in epidermal differentiation in a stage-specific manner. The potential of PHBV nanofibers in promoting BM-MSC proliferation was demonstrated through an MTS assay. In particular, BM-MSC proliferation in induction media on PHBV nanofibers was significantly higher compared to cells cultured on tissue culture polystyrene (TCPS) or PHBV with normal media.

The electrospun polycaprolactone (PCL)-polyethylene glycol (PEG) (PCL:PEG = 90:10, *w*/*w*) scaffolds, incorporating emu oil (EO), a natural substance with wound healing properties, were fabricated in the study by Pilehvar-Soltanahmadi et al. [41]. The field emission scanning electron microscopy (FE-SEM) images showed that the nanofibers had a smooth surface with diameters in the range of 150–300 nm. The addition of EO did not significantly alter the fiber diameter distribution, while EO-loaded scaffolds showed significantly higher tensile strength (5.8 MPa) compared to the PCL/PEG scaffolds (3.7 MPa). Other analyses showed that adipose-derived mesenchymal stem cells (Ad-MSCs) adhered to and proliferated more effectively on the EO-blended nanofibers compared to the PCL/PEG nanofibers. A cytoprotective effect of the scaffold against cell-damaging free radicals was also discovered, as demonstrated by a radical scavenging assay. Furthermore, immunostaining and gene expression analyses showed upregulation of keratin 10, filaggrin, and involucrin in Ad-MSCs cultured on the scaffolds in the presence of an epidermal induction medium consisting of DMEM and Ham’s F12 medium (3:1), supplemented with 10% FBS, 100 IU/mL penicillin, 100 µg/mL streptomycin, 0.4 µg/mL hydrocortisone, 5 µg/mL insulin, 1 nM T3, 10 ng/mL EGF, 1 µM VD3, and 50 µg/mL L-AA. Another attempt to induce epithelial differentiation starting from Ad-MSCs was carried out in the study by Javad Mirzaei-Parsa et al. [42]. Ad-MSCs were seeded on electrospun nanofibrous polycaprolactone (PCL)/fibrinogen (Fbg) scaffolds using a DMEM/F12 epidermal induction medium supplemented with FBS (5%), 1× insulin–transferrin–selenium (ITS), 0.5 μg/mL hydrocortisone, 10 ng/mL EGF, 10 ng/mL KGF, and 50 μg/mL L-ascorbic acid. Blending fibrinogen with PCL has been shown to improve the hydrophilicity and cytocompatibility of PCL nanofibers, leading to enhanced cell adhesion and proliferation. Differentiation was confirmed by the positive expression of keratin 10, filaggrin, and involucrin in a stage-specific manner. Skin wounds can be categorized as superficial, partial-thickness, or full-thickness wounds, with the latter category being more difficult to treat due to the lack of dermal tissue; to address this, an acellular dermal matrix (ADM) derived from rat skin dermal extracellular matrix (ECM) was used in this study. In animal studies, full-thickness excisional wounds were created on the backs of rats and treated with different groups, including ADM, ADM Ad-MSCs, nanofiber, nanofiber-Ad-MSCs, bilayer, and bilayer Ad-MSCs. Among these experimental groups, nanofiber-Ad-MSCs showed the most favorable outcomes in terms of re-epithelialization, average blood vessel density (92.7 ± 6.8), and collagen density (87.4 ± 4.9%).

In the study by Bhowmick et al., the synergistic effect of an electrospun nanofibrous composite scaffold made of cationic gelatin, hyaluronan, and chondroitin sulfate, which was loaded with sericin, was investigated [43]. Mechanical property analyses showed that the sericin-loaded scaffold exhibited a 79.1% increase in Young’s modulus (YM) and a 93.24% increase in maximum force at break compared to the native scaffold. The impact of a contact co-culture of human mesenchymal stem cells (hMSCs) and keratinocytes on the differentiation of hMSCs toward the epithelial lineage was also examined. After five days of contact co-culture, the results revealed that the electrospun scaffold containing sericin promoted the epithelial differentiation of hMSCs. This was evidenced by the expression of several marker proteins associated with epithelial cells (keratin 14, ∆Np63α, and pan-cytokeratin) and the upregulation of certain dermal proteins (keratin 14, ∆Np63α) at the gene expression level. These results confirmed that the sericin-loaded scaffold and the co-culture of keratinocytes and hMSCs synergistically stimulated the differentiation of hMSCs toward the epithelial lineage. In another study by Bhowmick, electrospun nanofibrous scaffolds made of gelatin/sulfated hyaluronan (sHA) or native hyaluronan (HA)/chondroitin sulfate (CS) were used in combination with keratinocytes (HaCaT) and hMSCs in a contact co-culture [44]. In this case, it was demonstrated that negatively charged sulfated glycosaminoglycans (GAGs) had a similar effect on promoting epithelial differentiation compared to sericin. The co-culture of hMSCs with keratinocytes on electrospun scaffolds containing sHA and CS resulted in the cellular fusion of the majority of hMSCs. In non-fused hMSCs, an increase in several epithelial markers was observed, suggesting enhanced epithelial differentiation.

Gelatin (20%wt) was also employed to prepare electrospun PCL/gelatin nanofibers loaded with 5%, 10%, and 15% manganese nanoparticles (MnO_2_–NPs) [45]. Rat adipose-derived stem cells (Ad-MSCs) were seeded on PCL/Gel-MnO2-NPs nanofibers to investigate the second-degree burn wound treatment in rats. The mechanical testing results indicate that nanofibers without nanoparticles exhibit high elongation, low ultimate tensile strength (UTS), and significant elasticity. However, the incorporation of MnO_2_ nanoparticles alters these properties. In nanofibers containing nanoparticles, both elasticity and elongation percentage are reduced, while the UTS value shows a notable increase that is directly proportional to the MnO_2_–NP concentration. This study demonstrated that a dressing made of PCL/gel combined with 5% MnO_2_-NPs and Ad-MSCs effectively promotes burn wound healing. It achieves this by enhancing collagen production, increasing GAG content, accelerating wound closure, and reducing levels of IL-1, IL-6, and α-SMA, thereby minimizing scar formation and inducing Ad-MSCs toward gradual epithelial integration and differentiation.

PCL and gelatin, as base materials, were also used by Majidansari et al. to produce a PCL/poly (glycerol sebacate) (PGS)/GEL nanofibrous scaffold with appropriate mechanical properties to support endothelial basement membrane development [46]. Ad-MSCs were seeded on fibers, and immunofluorescence studies and gene expression were performed, confirming that the electrospun PCL/PGS/gelatin scaffold significantly enhances the expression of endothelial-specific genes when growth factors are present in the cell culture medium.

The study by Ribeiro et al. focused on the development of a composite matrix for skin regeneration, which was produced by electrospinning collagen and electrospraying nanophased hydroxyapatite (nanoHA) [47]. The researchers aimed to evaluate the effect of nanoHA, as a localized calcium source, on the growth, proliferation, differentiation, and extracellular matrix production of human dermal fibroblasts, keratinocytes, and human mesenchymal stem cells (hMSCs). The results demonstrated that calcium ions released by nanoHA significantly boosted cellular growth and proliferation rates. Additionally, nanoHA inhibited the adhesion of pathogenic bacterial strains commonly found in human skin flora. In terms of mechanical properties, the composite mats exhibited remarkable tensile strength (428 ± 54 kPa). A preliminary in vivo study involving subcutaneous implantation of the matrix showed no adverse reactions from the host organism and indicated potential for de novo vascularization. Based on these findings, the composite membranes can serve for guided skin regeneration, providing a supportive and antimicrobial environment for wound healing.

A skin substitute was optimized by Steffens et al., integrating mesenchymal stem cells, keratinocytes, and an electrospun biomaterial [48]. Three typologies of electrospun scaffolds were prepared based on (1) poly-dl-lactic acid (PDLLA); (2) hydrolyzed PDLLA (PDLLA/NaOH); and (3) PDLLA/Lam—a PDLLA/NaOH scaffold linked to laminin (Lam) protein. PDLLA/Lam—a PDLLA/NaOH scaffold—showed the greatest hydrophilic characteristics and the best results concerning cell adhesion and viability.

In the field of research on predictive models for cancer study, polyvinyl alcohol (PVA)/silk sericin (SS) nanofibers (with an average diameter of 200 nm) were used to investigate the cell–nanomaterial interaction during the epithelial–mesenchymal transition of A549 cells [49]. Since electrospun PVA and PVA/SS fibers dissolve rapidly in water, they were crosslinked before being used as a cell culture substrate. The matrix was immersed in a crosslinking solution (comprising 5 mL of 50% glutaraldehyde, 5 mL of 37% HCl, and 40 mL of 100% ethanol) at room temperature for 6 h. Following this, it was rinsed twice with sterile water and soaked overnight in a 5 wt% glycine solution to detoxify the fibers and ensure complete removal of glutaraldehyde prior to cell culture. Researchers’ findings revealed that culturing A549 cells on a nanofibrous mat enhances their epithelial–mesenchymal transition (EMT) rate compared to cells cultured on a standard dish. This suggests that the nanofiber topology may contribute to an increased metastatic potential of A549 cancer cells.

Table 2 shows examples of epithelial differentiation induced on nanofibrous scaffolds specifying the type of nanofibers, the mechanical properties, the source of MSCs and the additional factors used to induce the differentiation.

### 2.3. Myogenic Differentiation

Many studies on myogenic differentiation from stem cells using electrospun scaffolds have primarily focused on the development of bladder smooth muscle cells (SMCs). The pursuit of innovative bladder tissue engineering solutions is critical due to the complications associated with conventional treatments like enterocystoplasty [50]. Such complications can include metabolic imbalances, urolithiasis, increased mucus production, infections, and even malignancies. Bladder dysfunction can arise from conditions like diabetes, Parkinson’s disease, stroke, and multiple sclerosis, often resulting in disrupted muscle layer organization, frequent infections, urinary incontinence, retention, voiding difficulties, and persistent muscle weakness.

**Figure 4:**
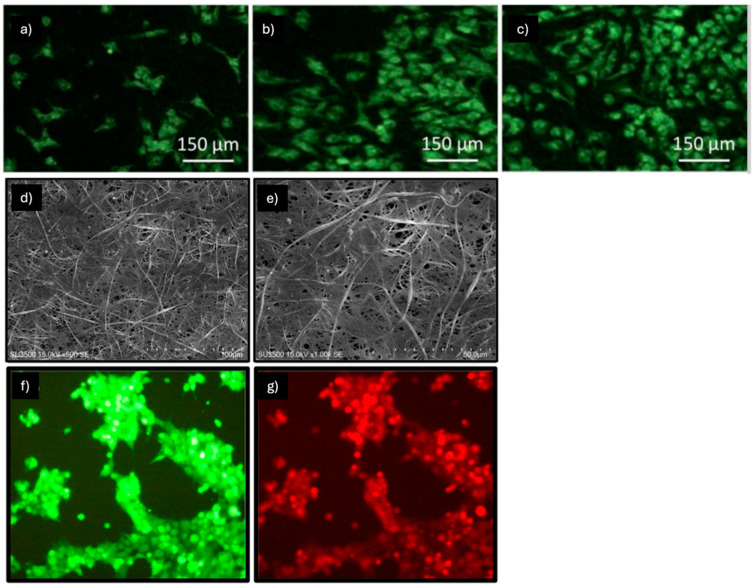
(**a**–**c**) Immunocytochemistry assay results for bladder myosin heavy chain (MHC) protein in human adipose-derived mesenchymal stem cells (AT-MSCs) cultured on (**a**) tissue culture polystyrene (TCPS); (**b**) poly lactic-co-glycolic acid (PLGA)/polyurethane (PU); (**c**) poly-phosphate (poly-P)-incorporated PLGA/PU nanofibrous scaffold (PLGA/PU/poly-P). Images obtained by [51]. (**d**,**e**) SEM images of human AT-MSCs seeded on electrospun PAN-PEO nanofibers. Immunocytochemistry assay of (**f**) bladder myosin heavy chain (MHC) and (**g**) α-SMA protein in smooth muscle cell (SMC)-differentiated human AT-MSCs grown on PAN-PEO nanofibers. Images obtained by [52].

Hossein Rezaei et al. conducted a study using electrospun poly(lactic-co-glycolic acid)-polyurethane (PLGA-PU) nanofibrous scaffolds, both with and without poly-phosphate (poly-P); in particular, the study investigated the possible role of poly-P in supporting the bladder smooth muscle cell (SMC) differentiation potential of human adipose-derived mesenchymal stem cells (Ad-MSCs) (Figure 4a–c) [51]. The use of Poly-P, which can be released continuously from the nanofibers, can trigger the mTOR pathway through PI3K/AKT activation, enhancing the efficacy of SMC differentiation, since mTOR and AKT pathways are both involved in SMC modulation. The differentiation of Ad-MSCs was analyzed by supplementing the basal medium (DMEM/10% FBS) with AA (30 mM) and TGF-β1 (5 ng/mL). The results obtained from this study demonstrate a greater SMC supportive capacity when Ad-MSCs were cultured on PLGA/PU scaffolds compared to the TCPS used as a control.

Analysis by RT-PCR after polyP incorporation into the PLGA/PU nanofibers shows increased gene expression of SMC-related gene markers, such as smooth muscle 22 alpha (SM-22a), alpha-smooth muscle actin (ASMA), calponin 1, myosin heavy chain (MHC), and Col-I. Gene expression on PLGA/PU/poly-P scaffolds was significantly higher at 7 and 14 days after differentiation induction compared to the PLGA/PU and TCPS groups. Also, the protein expression analysis via immunocytochemistry (ICC) of bladder MHC demonstrated increased expression on PLGA/PU/poly-P scaffolds. Therefore, PLGA–PU scaffolds, with or without poly-P, are promising for supporting human MSC differentiation into smooth muscle cells for bladder regeneration.

Another attempt to improve bladder smooth muscle cell differentiation from human Ad-MSCs was conducted in the study by Maryam Fakhrieh et al. using an electrospun polyacrylonitrile/polyethylene oxide nanofibrous scaffold (PAN-PEO) (Figure 4d–g) [52]. PAN-PEO electrospun scaffolds have fibers with average diameters ranging from 390 to 130 nm and exhibit good biocompatibility, along with suitable mechanical properties for soft tissue engineering. Following the assessment of the scaffold’s mechanical properties through a tensile test, peak stress was found to be 2.14 ± 0.23 MPa, break strain reached 28.42 ± 2.12 (%), and Young’s modulus stood at 109.42 ± 4.55 MPa. In a previous study, SMC differentiation was induced using a basal medium supplemented with 5 ng/mL TGFβ1 and 30 mM AA. The reported results from gene expression analysis via RT-PCR of α-SMA, SM22α, calponin1, caldesmon1, and MHC in Ad-MSCs in differentiation medium cultured on PAN-PEO were significantly higher than those of Ad-MSCs cultured on TCPS, which was used as the control. Protein expression analysis by ICC of α-SMA and MHC, considered early and late differentiation markers, respectively, was also conducted; both of these markers were significantly more expressed in AT-MSCs cultured on PAN-PEO than in stem cells cultured on TCPS.

Non-thermal oxygen plasma modified polyaniline (PANi)/polyacrylonitrile (PAN) conductive electrospun nanofibers were prepared to induce human bone marrow-derived mesenchymal stem cells to differentiate into muscle-like cells [53]. After 21 days of muscle differentiation induction, the expression of the α-actinin, myosin, and myogenin genes was confirmed using reverse transcription polymerase chain reaction, while the presence of M-cadherin and troponin was validated through immunocytochemistry.

Poly(vinylidene fluoride) (PVDF) nanofibrous scaffolds were fabricated using the electrospinning method, with or without chitosan nanoparticles loaded with TGFβ [54]. Adipose tissue-derived mesenchymal stem cells (Ad-MSCs) were isolated and characterized immediately, and smooth muscle cell (SMC) differentiation was studied when they were cultured on the scaffold surface.

An electrospun poly(lactic-co-glycolic acid) (PLGA) nanofibrous scaffold was used in the study conducted by Ali Mirzaei et al. in order to induce SMC differentiation from human-induced pluripotent stem cells (hiPSCs) [55]. Morphological analysis via SEM showed a fibrous scaffold characterized by smooth, bead-free, and randomly oriented fibers with interconnected pores and nanometer sizes averaging 600 ± 400 nm; mechanical characterizations showed a tensile strength of 11.32 ± 2.02 MPa and an elongation at break of 32.43 ± 1.97%. After the preparation of hiPSCs embryonic bodies (EBs), 50 EBs per well (24-well plate) were transferred to the PLGA nanofibrous scaffolds and TCPS as a control and incubated in differentiation medium composed of DMEM supplemented with 5% FBS, 5 ng/mL TGFβ1, and 30 mM AA for three weeks. Again, successful differentiation was confirmed by the overexpression of SM22-a, Calpinin1, Caldesmin1, and MHC genes in human iPSCs, as shown via RT-PCR analysis; the expression of these genes was higher in cells grown on scaffolds than those grown on TPCS.

As a new approach in the field of bladder tissue engineering, an electrospun PCL (containing TGF-β1-loaded chitosan nanoparticles)-PLLA scaffold, denoted as PCL/NP(TGF)-PLLA meshes, was proposed in order to induce SMC differentiation from human Wharton’s jelly-derived umbilical cord mesenchymal stem cells (hWJ-derived UC-MSCs) [56]. In this study conducted by Samaneh Moghadasi Boroujeni et al., it was observed that the sustained release of encapsulated TGF- β1 for more than 10 days can enhance the myogenic differentiation potential of UC-MSCs. Starting from the assumption that TGFβ1 is the most potent inducer of MSC myogenic differentiation, its encapsulation helped maintain its activity despite its well-known short serum half-life. The chitosan particles were distributed in the nanofiber core, and this allowed for a slow and sustained release profile of TGF-β1, which provided a long-term biochemical signal to the seeded cells. The release of TGF-β1 from the PCL/NP(TGF)-PLLA nanofibrous scaffold occurred through two main mechanisms, including chitosan degradation and diffusion through the PCL nanopores.

Wang et al. also obtained a multilayered nanofibrous patch functionalized with adipose tissue extract (ATE) for the treatment of bladder regeneration [57]. ATE is a non-cellular three-dimensional macromolecular network that can influence the local microenvironment for cells, affecting cellular metabolism, proliferation, differentiation, and organoid formation. PLA-PCL electrospun nanofibers were composed into a multilayered patch with ATE/hyaluronic acid (HA) gel solution to obtain a five-layer flat patch. The patches were then frozen at −20 °C for 24 h and then vacuum-dried for 48 h. This hierarchical structure, which combines various physical and biochemical cues, plays a crucial role in vitro and in vivo in influencing cell behaviors, including attachment, proliferation, and differentiation, thereby significantly impacting bladder reconstruction and cellular responses.

Heparin-conjugated polycaprolactone (HPCL) nanofibers were developed by Awadalla et al. to study the differentiation of AD-MSCs into smooth muscle cells (SMCs) as a promising treatment for urinary tract injury [58]. Heparin also demonstrated a role in preventing blood clotting and enhanced the compatibility of HPCL coatings with physiological tissues. Ad-MSCs cultured on the HPCL scaffold showed a significant increase in the expression of specific proteins compared to those cultured on the PCL scaffold (*p* < 0.05). Additionally, SMCs on PCL scaffolds exhibited higher levels of MHC and α-SMA compared to Ad-MSCs on both PCL and HPCL scaffolds (*p* < 0.05). Notably, SMCs cultured on the HPCL scaffold demonstrated the highest protein concentrations among all groups.

SMC differentiation of UC-MSCs was evaluated under three different culture conditions: on TCP with basal medium supplemented with 2.5 ng/mL TGF-β1 and 30 mM AA, on PCL-PLLA scaffolds with the same differentiation medium, and to assess the effect of controlled TGF-β1 release, on PCL/NP(TGF)-PLLA scaffolds treated with basal medium supplemented only with 30 mM AA. RT-PCR analyses confirmed that Calponin1 and SM22α gene expression was significantly higher in cells cultured on scaffolds, both PCL-PLLA and PCL/NP(TGF)-PLLA, than in those on TCP. Furthermore, although TGF-β1 was present in much lower concentrations (>10 times lower on average) in PCL/NP(TGF)-PLLA scaffolds than in the other two conditions with bolus delivery, a marked increase in ASMA gene expression was observed. Immunofluorescence staining for ASMA and Desmin after 15 days of culture showed higher intensity for cells seeded on PCL/NP(TGF)-PLLA than on TCP and PCL-PLLA scaffolds, demonstrating that PCL/NP(TGF)-PLLA scaffolds promote the SMC differentiation of MSCs more significantly.

Myogenic differentiation starting from MSCs alone might not always be satisfactory; for this reason, in the study by R. Witt et al., rat MSCs isolated from bone marrow were co-cultured with rat myoblasts. It is well known that MSCs can fuse with myoblasts and contribute to the muscle regeneration process. Additionally, they stimulate myoblast migration, proliferation, and differentiation by releasing factors involved in muscle regeneration, such as basic fibroblast growth factor (bFGF), hepatocyte growth factor (HGF), and insulin-like growth factor 1 (IGF-1) [59]. One of the aims of the study was to evaluate myogenic differentiation in mono- and co-cultures of myoblasts and MSCs using different concentrations of HGF and IGF-1, which were supplemented with a basic differentiation medium (DMEM/Ham’s F-12) composed of 2% donor horse serum (DHS), 1% L-glutamin, 1% penicillin/streptomycin (P/S), 0.4 μg/mL dexamethasone (DXM), and 1 ng/mL bFGF. Based on the premise that a parallel alignment of fibers could stimulate myotube formation and that the combination of PCL and collagen provides strength, elasticity, and compliance, a parallel-aligned electrospun PCL-collagen-I scaffold seeded with MSCs and myoblasts at a 1:1 ratio was used; in this case, differentiation was induced with the abovementioned medium characterized by a combination of HGF (10 ng/mL) and IGF-1 (10 ng/mL) for 7 days. SEM analyses demonstrated the attachment, proliferation, and parallel alignment of MSCs + myoblasts on the scaffolds. Furthermore, positive staining for Desmin was observed, which correlated with efficient myogenic differentiation. Therefore, this scaffold represents a promising alternative by mimicking the structure of skeletal muscle and promoting the parallel alignment of MSCs and myoblasts.

A development of the reported study is described in the work of Aijia Cai et al., in which the same electrospun PCL-collagen-I scaffold was used for the co-culture of primary myoblasts (Mb) and mesenchymal stromal cells derived from rat BM-MSCs and Ad-MSCs [60]. Since the use of serum can lead to heterogeneous results due to variations in different serum lots and safety concerns, several groups have focused on the use of serum-free media. In this case, differentiation on the scaffolds was induced using a serum-free myogenic differentiation medium chosen after it yielded better results than two other serum-free media in mono- and co-cultures under 2D conditions; this medium contained 1% L-glutamin, 1% P/S, 0.4 μg/mL DXM, and 1 ng/mL bFGF, supplemented with DMEM/Ham’s F12 + 0.2% Ultroser^®^ G. The results obtained demonstrate that serum-free myogenic differentiation is feasible; better expression of some major related myogenic differentiation genes was observed in myoblast/Ad-MSC co-cultures compared with those with BM-MSCs. After 28 days from differentiation induction, it was possible to observe multinucleated cells via fluorescence microscopy and, therefore, the possible formation of myotubes in the serum-free co-cultures when compared under the same conditions in medium containing 2% DHS. Furthermore, the positive expression of myosin heavy chain for BMSC/Mb and Ad-MSC/Mb was observed.

A recent study by Aijia Cai et al. builds on the two previous works with the aim of co-culturing Schwann cells (SCs) with Mb/Ad-MSC on an electrospun PCL-collagen-I scaffold to evaluate whether SCs, as part of the peripheral nervous system, could promote myogenic differentiation given their ability to enhance synaptic junction formation [61]. The myogenic differentiation medium used contained DMEM/Ham’s F12 + 2% DHS + 1% L-glutamine + 1% P/S + 0.4 µg/mL DXM and 1 ng/mL bFGF. Mb/Ad-MSC/SC (1:1:0.5) were co-cultured on PCL-collagen I nanoscaffolds and myogenically differentiated for 28 days. The co-culture of Mb/Ad-MSC (1:1) was seeded under the same conditions to evaluate whether any differences existed after adding the SCs. The results obtained indicated greater gene expression of myosin heavy chain 2 (MYH2) and myogenin (MYOG) in Mb/Ad-MSC/SC compared to Mb/Ad-MSC; however, no significant difference was observed in the gene expression of skeletal alpha actin 1 (ACTA1). Furthermore, fluorescence microscopy analysis detected a significant and clearer number of aligned multinucleated cells in the Mb/Ad-MSC/SC, suggesting possible myotube formation. In conclusion, the study shows how SC inclusion can promote the myogenic differentiation of co-cultured primary human Mbs and Ad-MSCs.

In a more recent study, aligned silk fibroin/Fe3O4 nanoparticles-blend (SF/Fe) nanofiber scaffolds were obtained by integrating a magnetic field collection device and incorporating Fe_3_O_4_ nanoparticles into the spinning solution [62]. These aligned nanofibers (730 nm) demonstrated high tensile strength and elasticity modulus along their orientation direction. Moreover, the aligned SF/Fe scaffolds effectively guided the adhesion, proliferation, and differentiation of mesenchymal stem cells along the fiber direction. Myoblast C2C12 cells cultured on these scaffolds exhibited oriented growth, emphasizing the potential of aligned SF/Fe fibers in advancing skeletal muscle engineering for biomedical applications.

Wang et al. obtained the differentiation of iPSC-derived mesenchymal stem cells (iMSC) into mature, contractile smooth muscle cells (SMCs) [63]. Electrospun α-amino acid-substituted poly(organophosphazene) fibers (~200 nm in diameter) were obtained. PαAPz-F was derived from the amino acid L-phenylalanine, while PαAPz-A was derived from L-alanine. The results obtained demonstrated that electrospun fibers from PαAPz-A were able to support adhesion and spreading of iMSC, BM-MSC, and primary human coronary artery SMC; moreover, they were able to promote the differentiation of iMSCs toward the SMC lineage.

Table 3 shows examples of myogenic differentiation induced on nanofibrous scaffolds specifying the type of nanofibers, the mechanical properties, the source of MSCs and the additional factors used to induce the differentiation.

### 2.4. Tendon Differentiation

Tendon injuries present a significant clinical challenge, particularly because tendons have a limited ability to regenerate. These injuries often occur in sports or other physically demanding activities, necessitating effective treatment strategies. Conventional approaches, such as autografts, allografts, xenografts, and prosthetic devices, while commonly used, have limitations. These include issues like donor site morbidity, risks of disease transmission, and compromised long-term functionality. In light of these challenges, tendon tissue engineering has emerged as a promising avenue for addressing tendon injuries, garnering growing interest due to its potential to improve treatment outcomes [64].

The study conducted by Zhou et al. investigated the effects of microscaled and nanoscaled aligned topography on inducing tenogenic (tendon-like) differentiation of human adipose tissue-derived mesenchymal stem cells (hAd-MSCs) using parallel microgrooved PDMS membranes and parallel-aligned electrospun nanofibers made from a gelatin (GEL)/poly-ε-caprolactone (PCL) mixture [65]. The expression of various tenogenic markers, including tenomodulin, collagen I, collagen VI, decorin, tenascin-C, and biglycan, was induced by aligned topography at both scales. However, the upregulated expression of scleraxis and tenascin-C was observed exclusively in the microscaled topography. In addition, tenogenic differentiation was confirmed on the third day of culture only in microscaled topography. Neotendon tissue with mature type I collagen fibers was formed in vitro when mouse ASCs were cultured on parallel-aligned polyglycolic acid (PGA) microfibers; in contrast, randomly patterned PGA microfibers resulted in the formation of fat tissue.

Xing Li et al. fabricated and characterized photo-crosslinked polycaprolactone/methacrylated poly(trimethylene carbonate) (PCL/PTMC-MA) composite electrospun scaffolds, incorporating PTMC-MA into PCL [66]. The mechanical analysis showed that as the content of PTMC-MA increased, the mechanical properties of the PCL/PTMC-MA composite scaffolds improved. After photo-crosslinking, the scaffolds exhibited enhanced mechanical properties, including superior creep resistance, compared to pure PCL scaffolds. Notably, the photo-crosslinked PCL/PTMC-MA (1:3) composite scaffold demonstrated a Young’s modulus of 31.13 ± 1.30 MPa and a maximum stress at break of 23.80 ± 3.44 MPa, which were comparable to the mechanical properties of native tendon. Biological experiments using MSCs demonstrated that the PCL/PTMC-MA composite scaffolds were biocompatible, supporting cell adhesion, proliferation, and differentiation.

**Figure 5:**
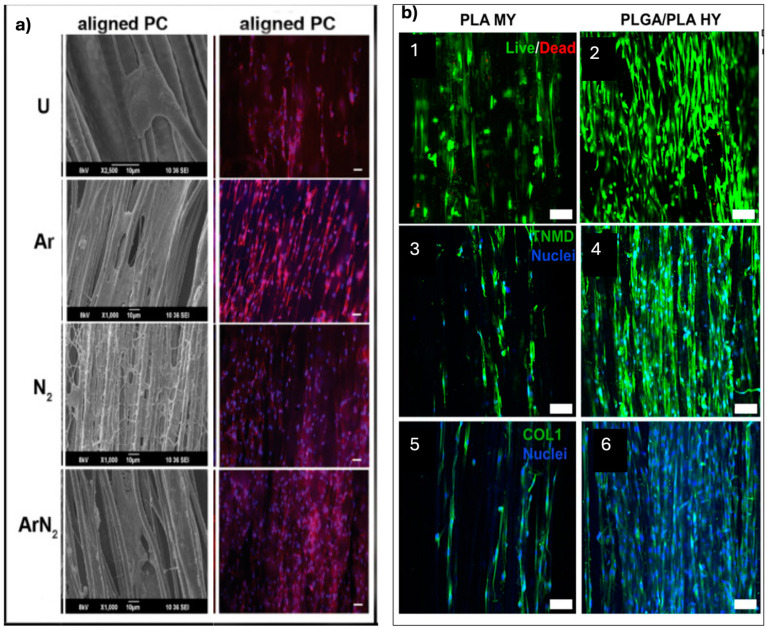
(**a**) SEM images showing cell adhesion within 4 h of culture and fluorescent microscopy images of actin and nucleus-stained cells after 24 h of culture on plasma-treated and untreated aligned PCL/collagen multiscale fibers (PC). The scale bar for SEM images is 10 μm, and for fluorescent microscopy images, it is 50 μm. Images sourced from [67]. (**b**) (1, 2) Representative fluorescent images showing living cells (green) and dead cells (red) of HADMSC seeded on PLA MY bundles and PLGA/PLA HY bundles conditioned in TM for 21 days. (3, 4) Immunofluorescence (IF) staining for TNMD (green) and nuclei (blue) of HADMSC after 21 days of tenogenic differentiation. (5, 6) IF staining for COL1 (green) and nuclei (blue) of HADMSC after 21 days of tenogenic differentiation. Scale bar: 100 μm. Images sourced from [70].

Sankar et al. conducted a study focusing on developing aligned poly(ε-caprolactone)/collagen (PCL/Collagen) multiscale fibers as scaffolds for tendon tissue engineering; in particular, a plasma treatment using argon, nitrogen, or a combination of both gases was applied to the fibers with the aim of inducing tenogenic differentiation in MSCs seeded on the scaffolds (Figure 5a) [67]. The results showed that plasma treatment increased the hydrophilicity of the fibers by incorporating polar functional groups. In addition, the nanoroughness was enhanced without compromising structural integrity. Enhanced proliferation, attachment, infiltration, and elongation were observed in MSCs on the plasma-treated fibers; this was attributed to the activation of the RhoA signaling pathway, which is known to play a role in tenogenic differentiation. Furthermore, the expression of various markers associated with tenogenesis was increased at the protein (scleraxis, mohawk, tenomodulin) and gene (mohawk, collagen I, collagen III, thrombospondin 4, tenascin-C) levels on argon plasma-treated fibers. Overall, the findings suggest that argon plasma treatment of aligned PCL/collagen multiscale fibers can effectively induce tenogenic differentiation in MSCs, even in non-tenogenic media.

To examine the effect of scaffold design and polymer type on the mechanical properties and tenogenic differentiation of seeded MSCs, Rothrauff et al. developed four scaffolds: (1) braided PCL, (2) braided PLLA, (3) stacked PCL, and (4) stacked PLLA [68]. Human BM-MSCs were cultured on various scaffolds for up to 28 days, with assessments made on tenogenic differentiation, histological features, and biochemical composition. The braided scaffolds demonstrated superior tensile and suture-retention strengths, although their elastic moduli were lower. Both types of scaffolds promoted the expression of tenogenic markers, with the braided scaffolds showing a more pronounced effect. On the other hand, the stacked scaffolds allowed for greater cell infiltration, resulting in higher cell counts, collagen content, and sulfated glycosaminoglycan levels. However, when adjusted for cell number, both scaffold designs exhibited similar abilities to influence extracellular matrix protein deposition. This study underscores the importance of scaffold macro-architecture, particularly multilayered scaffolds made from aligned electrospun nanofibers, in supporting the tenogenic differentiation of MSCs for tendon and ligament tissue engineering applications.

A novel approach was developed in the study by Wu et al. to create nanofibrous woven biotextiles for tissue-engineered tendon scaffolds [69]. The process involved fabricating polycaprolactone (PCL) nanofiber yarns using a modified electrospinning setup. These nanofiber yarns were then woven into fabrics interlaced with PLA multifilaments. Compared to non-woven nanofibrous structures (random and aligned mesh) generated using traditional electrospinning, the woven fabrics exhibited distinct advantages, such as 3D aligned microstructures, larger pore sizes, and significantly improved tensile mechanical properties. The tensile strength of woven fabrics aligned in the parallel direction of PCL nanofiber yarns was approximately five times greater than that of random nanofibrous scaffolds with random orientation. Similarly, the Young’s modulus of the aligned woven fabrics was over ten times higher. Additionally, the tensile strength of the woven fabrics along the aligned direction was more than twice as high, and the Young’s modulus value was approximately five times higher compared to the aligned nanofibrous scaffolds. These features were beneficial for cell proliferation, infiltration, and the expression of tendon-specific genes by hTCs and hAd-MSC after their culture in a tenogenesis induction medium containing DMEM/F12 medium, 2% FBS, and 20 ng/mL TGF-β3. Furthermore, co-cultures of hAd-MSC with hTCs or hUVEC on the woven fabrics demonstrated enhanced expression of tenogenic markers. The highest upregulation of tendon-associated markers was observed in Ad-MSC/hTCs/hUVEC tri-cultures on the woven fabrics, indicating the synergistic effect of multiple cell interactions. Additionally, the study explored the dynamic conditioning of the tri-cultured constructs and found that dynamic stretch further promoted collagen secretion and tenogenic differentiation.

In another study, Wu et al. used a modified electrospinning device to coat PLGA nanofibers onto PLA microfiber yarns (MY), creating PLGA/PLA hybrid yarns (HY) for tendon tissue engineering applications (Figure 5b) [70]. The PLGA/PLA HY exhibited a well-aligned nanofibrous structure that resembled the ultrastructure of native tendon tissues. The hybrid yarns showed an enhanced failure load compared to PLA MY, indicating improved mechanical properties, as well as superior cell interactions. HAd-MSCs cultured under the same tenogenesis induction medium on the hybrid yarns exhibited enhanced growth, proliferation, and tendon-specific gene expression, suggesting a favorable microenvironment for tenogenic differentiation. In addition, thymosin β4 (Tβ4), a bioactive protein factor involved in tendon regeneration, was encapsulated within the PLGA nanofibers during the electrospinning process. The Tβ4-loaded PLGA/PLA HY exhibited sustained drug release for 28 days and exerted additive effects on hAd-MSC behaviors, promoting cell migration, proliferation, and tenogenic differentiation.

Another attempt to resemble the structure of native tendons was carried out by Wu et al., who fabricated composite electrospun scaffolds of poly(p-dioxanone) (PPDO) and silk fibroin (SF), followed by thermal ethanol treatment [71]. The PPDO/SF composite scaffolds exhibited a parallel fiber arrangement with crimped features, including nonlinear behavior that could be adjusted by varying the mass ratio of PPDO/SF; several different wavy nanofibrous scaffolds (WNSs) were fabricated at various ratios of PPDO and SF (4:1/2:1). This allowed for the tunability of the fiber crimp degree and mechanical properties to better mimic the native tendon tissue mechanics. Biological tests demonstrated that the addition of SF to the scaffolds improved cell adhesion, proliferation, and phenotypic maintenance of human tenocytes (hTCs) on the scaffolds. The PPDO/SF scaffolds also showed a decrease in inflammatory response compared to pure PPDO scaffolds when implanted subcutaneously. In addition, hAd-MSCs were seeded on 4/1 PPDO/SF WNSs using the tenogenesis induction medium used in previous studies. Furthermore, to provide mechanical stimulation, a homemade bioreactor was used to apply cyclic stretching to the hAd-MSC-seeded PPDO/SF WNSs. In conclusion, the combination of growth factor induction and mechanical stimulation was found to enhance tenogenic differentiation.

Sarikaya et al. also utilized silk fibroin in combination with poly-3-hydroxybutyrate (SF/P3HB) to create scaffolds with aligned topography. The average fiber diameter was 699 ± 203 nm, and 80% of the nanofibers were aligned within a ±15° range [72]. The addition of SF reduced the crystallinity of P3HB, and the elastic modulus was measured at 197.0 ± 7.7 MPa. A 21-day cell culture study was conducted with rat rAd-MSCs, both with and without the addition of the tenogenic differentiation factor-5 (GDF-5). The findings revealed that SF/P3HB scaffolds supported cell proliferation and enabled differentiation into tenocytes.

The study by Bosworth et al. focused on investigating the behavior of hMSCs seeded on electrospun yarns (PCL) and their response to dynamic tensile loading [73]. The results showed that the hMSCs adhered and proliferated on the electrospun yarns, regardless of the loading regime. Importantly, the yarns guided the seeded cell alignment, with the majority of cells orienting parallel to the underlying fiber direction. When exposed to dynamic tensile loading, the cell-seeded yarns exhibited a thicker cell layer on the exterior of the scaffold compared to those cultured statically. This suggests an increased rate of cell proliferation and/or matrix deposition in response to mechanical stimulation.

The tensile properties of the cell-seeded yarns also improved over time compared to acellular yarns, indicating the potential for enhanced mechanical strength; the ultimate tensile strength (Mpa) increased from about 15 MPa to 55 MPa, and the Young’s modulus increased from about 40 MPa to 100 Mpa. Furthermore, the loaded scaffolds exhibited the upregulation of key tendon-related genes, including collagen type I, indicating the differentiation of hMSCs toward a tendon lineage when subjected to mechanical stimulation.

In order to develop a scaffold mimicking the size, stiffness, and strength of human tissues while maintaining sufficient porosity for cellular infiltration, the RPM speed of the collecting mandrel setup during the electrospinning of PCL was varied in the study by Olvera et al. [74]. This variation allows for control over the degree of inter-fiber fusion or fiber welding; by increasing the fraction of unfused fibers, the flexural rigidity of the electrospun sheets is reduced, enabling fiber bundling into 3D scaffolds with dimensions similar to those of the human anterior cruciate ligament (ACL). The higher rotational velocities of the collecting mandrel resulted in increased mechanical properties such as Young’s modulus, yield stress, and yield strain of the fiber bundles. At 3500 RPM, the fiber bundles exhibited a Young’s modulus of 121.5 ± 3.8 MPa, a yield stress of 6.3 ± 0.09 MPa, and a yield strain of approximately 10%. In contrast, at 500 RPM, the Young’s modulus was 14.4 ± 0.8 MPa, the yield stress was 0.6 ± 0.03 MPa, and the yield strain was approximately 6%. MSCs seeded onto these scaffolds and stimulated with appropriate growth factors exhibited increased expression of genes associated with ligament or cartilage tissue. The ligament induction medium specifically consisted of high-glucose DMEM GlutaMAX, supplemented with 2% FBS, penicillin (100 U/mL), streptomycin (100 μg/mL), 50 μg/mL L-AA 2-phosphate, 0.25 μg/mL amphotericin B, and 100 ng/mL recombinant human CTGF.

Su et al. fabricated a highly interconnective graphene oxide-doped electrospun poly(lactide-co-glycolide acid) (GO-PLGA) nanofibrous membrane to develop integrative biomaterials for promoting functional tendon-to-bone integration since normal entheses (the junction between tendon and bone) are not fully reestablished after surgical repair [75]. In vitro evaluations demonstrated that the incorporation of GO into PLGA membranes accelerated the proliferation of bone marrow-derived mesenchymal stem cells (BMSCs) and enhanced their osteogenic differentiation. The GO-PLGA nanofibrous membrane provided a suitable microenvironment for BM-MSCs, promoting their growth and differentiation. To further assess the efficacy of the GO-PLGA membrane, rabbit models were established, and the membranes were used to augment rotator cuff repairs. Postoperative evaluation through micro-computed tomography, histology, and biomechanical testing revealed promising results. The local application of the GO-PLGA membrane between the tendon and the bone in the rabbit models promoted enthesis healing, leading to increased new bone and cartilage generation. Moreover, the collagen arrangement and biomechanical properties were improved compared to repairs using PLGA alone. In conclusion, the electrospun GO-PLGA fibrous membrane demonstrated effective regenerative properties for promoting tendon-to-bone enthesis healing.

Zhang et al. utilized a spinning approach called stable jet electrospinning (SJES) to fabricate continuous, well-aligned ultrafine fibers that mimic the microstructure and mechanical properties of native tendons in order to induce tenogenic differentiation starting from hiPSC-derived MSCs [76]. In particular, the fibers are composed of Ch (62.1 wt%), PLLA (20.7 wt%), GEL (10.3 wt%), and PEO (6.9 wt%). Compared to the random scaffold, the aligned scaffold exhibited notably superior mechanical properties, like tensile strength (2.43 ± 0.28 MPa vs. 14.23 ± 1.08 MPa) and Young’s modulus (74.46 ± 12.99 MPa vs. 325.01 ± 25.05 MPa). The differentiation of hiPSC-derived MSCs into tenocyte-like cells was achieved by activating mechano-signaling pathways through the topographic cues provided by the aligned fibers. Furthermore, an in situ tendon repair study was conducted using a rat Achilles tendon repair model and demonstrated that the aligned fiber scaffold combined with hiPSC-derived MSCs had a significant effect on improving the structural and mechanical properties of tendon injuries.

The study by Yin et al. aimed to investigate the impact of different topographic cues provided by aligned and randomly oriented PLLA scaffolds on the growth and differentiation of mesenchymal stem cells (MSCs) [77]. Similar to the previous study, it was seen, both in vitro and in vivo, that the aligned-oriented scaffold group displayed more mature tendon-like tissue formation; conversely, the randomly oriented scaffold group exhibited chondrogenesis and subsequent bone formation. Overall, the study revealed that the release of cytoskeletal tension played a role in modulating the divergent differentiation pathways on different substrate topographies.

In ACL reconstruction surgery, the use of hamstring tendon autografts is a common choice; however, a major drawback of autografts is their inefficient healing with the host bone tunnel, which can lead to surgical failure. To address this issue, Han et al. fabricated a biomimetic nanofibrous membrane aimed at improving tendon–bone integration [78]. The nanofibrous membrane consisted of a PCL electrospun membrane and a chitosan/hyaluronic acid (Ch/HA) multilayer film. Using a layer-by-layer (LbL) self-assembly method, functional Ch/HA multilayer films were applied to the surface of the PCL nanofibers. Stromal cell-derived factor-1α (SDF-1α) and bone morphogenetic protein-2 (BMP-2), both of which are crucial for bone regeneration, were incorporated into the nanofibrous PCL membrane (S + B@P) via LbL. S + B@P demonstrated enhanced BMSC migration and osteogenic differentiation compared to the BMP-2-only-loaded scaffold (B@P) in vitro. In a rabbit ACL injury model, S + B@P was used for ACL reconstruction. After 4 weeks, S + B@P showed minimal fibrous tissue, tight autograft–bone attachment, and clear interface integration. By 8 weeks, significant new bone formation was observed, improving biomechanical properties. In conclusion, the membrane facilitated cell proliferation, recruitment, osteogenic differentiation, and the recruitment of bone marrow-derived mesenchymal stem cells (BM-MSCs).

Table 4 reported examples of tendon differentiation induced on nanofibrous scaffolds specifying the type of nanofibers, the mechanical properties, the source of MSCs and the additional factors used to induce the differentiation.

### 2.5. Vascular Differentiation

Atherosclerosis and coronary arterial restenosis are leading causes of cardiovascular morbidity and mortality worldwide. A critical challenge in treating these conditions arises when patients lack suitable autologous vessels for replacement, often due to prior surgical removal, underlying vascular disease, or the inadequate size of available vessels. This limitation underscores the need for alternative strategies, such as advanced graft materials or tissue-engineered vascular substitutes, to address deficiencies in vascular replacement therapies. Various studies have explored innovative approaches to advance vascular tissue engineering and the development of vascular grafts that exploit the differentiation ability of MSCs and nanofibrous scaffolds [81].

**Figure 6:**
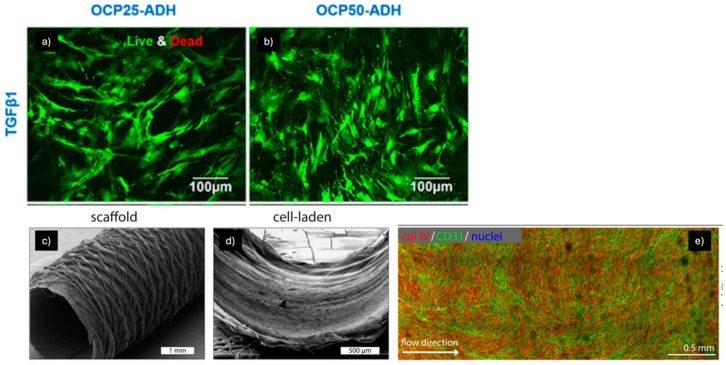
(**a**,**b**) Live/dead staining was performed to assess the viability of MSCs cultured on pectin nanofiber scaffolds with varying oxidation levels over a 14-day period. MSCs were cultured with TGFβ1 medium on (**a**) OCP25-ADH and (**b**) OCP50-ADH scaffolds to evaluate cell viability and proliferation, respectively. Images obtained by [82]. Scanning electron microscopy images of the PCL scaffold (**c**) without cells and (**d**) with cells. (**e**) An overview image showing cell coverage and alignment across the entire length of the perfused sample. Images obtained by [83].

For instance, Na Li et al. developed pectin hydrogel nanofiber scaffolds to mimic the mechanical properties and biocompatibility of native blood vessels (Figure 6a,b) [82]. These scaffolds, prepared with two oxidation degrees (25% and 50%) using periodate oxidation and ADH-mediated crosslinking, exhibited a nano-sized fibrous structure with diameters ranging from 300 to 400 nm, and their stiffness and hydrophobicity were modulated by adjusting the oxidation and crosslinking levels. Both types of scaffolds demonstrated high biocompatibility with mesenchymal stem cells (MSCs), which maintained high viability after 14 days. The mechanical properties of the scaffolds guided MSC differentiation: the stiffer scaffold (50% oxidation) promoted differentiation into vascular smooth muscle cells (vSMCs), while the softer scaffold encouraged differentiation into endothelial cells (ECs). These findings suggest that pectin hydrogel nanofibers can provide both mechanical support and differentiation cues, making them promising candidates for vascular tissue engineering and the development of functional vascular grafts.

In another study, performed by Iris Pennings et al., a bioreactor system was developed to culture bi-layered vascular grafts under shear stress, with compartmentalized exposure to cell-specific media for the luminal and outer layers of the graft (Figure 6c–e) [83]. The system enabled the co-culture of endothelial colony-forming cells and multipotent mesenchymal stromal cells (MSCs), produced by combining electrospinning and melt electrowriting techniques. The results showed that endothelial cells (ECs) and vascular smooth muscle cells (vSMCs) were successfully induced and maintained under flow perfusion. The endothelial layer exhibited flow responsiveness with the upregulation of key markers, while the MSCs in the outer layer differentiated into smooth muscle-like cells under TGFβ treatment. This bioreactor system allowed for simultaneous, layer-specific cell differentiation, successfully recapitulating the natural architecture and cell phenotypes of a native blood vessel, particularly the tunica intima and tunica media. The findings suggest that this co-culture system can be used to create and investigate vascular grafts from clinically relevant progenitor cell sources, offering a promising approach for advancing the next generation of bioengineered vascular grafts.

The study by Akshat Joshi et al. aimed to enhance the endothelization of artificial vascular grafts, particularly those with a diameter smaller than 5 mm, which are prone to thrombosis due to insufficient endothelization [84]. To address this, the authors co-cultured adipose-derived mesenchymal stem cells (Ad-MSCs) with human umbilical vein endothelial cells (hUVECs) on antithrombogenic polycaprolactone (PCL)/heparin-gelatin co-spun nanofibers. Using a coaxial electrospinning technique, they fabricated a scaffold that balanced mechanical properties with biocompatibility. Heparin was grafted onto the nanofibers to impart antithrombogenic properties. After performing the co-culture, the results showed enhanced CD-31 expression and healthy hUVEC morphology, indicating successful endothelization. The study concludes that co-culturing MSCs with hUVECs is an effective strategy for promoting rapid endothelization of vascular substrates without the need for surface modification using growth factors or other ECM proteins. This research highlights the effectiveness of heparinized co-spun PCL/gelatin nanofibers, combined with co-culture, in fabricating an ideal vascular graft. The nanofibers exhibited good mechanical properties, and surface modification with heparin provided the necessary antithrombogenic properties. The co-culture successfully promoted endothelization, forming a mature endothelial monolayer that fulfills the final prerequisite for an ideal graft.

In a study conducted by Muhammad Shafiq et al., cell-free, three-dimensional, bio-instructive small-diameter vascular grafts were developed using electrospinning techniques [85]. Heparin was conjugated to the scaffolds to suppress thrombogenic responses, and substance P (SP) was immobilized to recruit host cells. The sustained release of SP from the scaffolds was confirmed through ELISA, and in vitro cell migration assays showed successful recruitment of human mesenchymal stem cells (hMSCs). In vivo experiments revealed that grafts containing SP and heparin/SP demonstrated enhanced cell infiltration, MSC recruitment, blood vessel formation, and regeneration of smooth muscle cells (SMCs) and extracellular matrix (ECM) components compared to control grafts. These functionalized grafts also stimulated macrophage polarization into the M2 phenotype, which likely contributed to the regeneration of the cell-free grafts. This combinatorial biomaterial approach offers a promising strategy for designing next-generation vascular grafts by modulating host responses, promoting angiogenesis, and improving tissue regeneration. SP- and heparin-functionalized PLCL copolymers provide long-term, localized persistence of biomolecules in vivo, further enhancing the potential for vascular graft applications. Future research may focus on refining biomolecule immobilization and evaluating graft performance under circulation.

Yong-Chao Jiang et al. developed a scaffold platform designed to release growth factors, consisting of polycaprolactone (PCL) nanofibers combined with vascular endothelial growth factor (VEGF)-encapsulated gelatin particles. This platform was created to prolong the effects of growth factors on mesenchymal stem cells (MSCs) and endothelial cells (ECs), enhancing their proliferation and differentiation) [86]. The scaffold successfully directed MSC differentiation into ECs while maintaining the stability of the tubular structure of ECs, which is an indicator of angiogenesis. The results suggest that PCL/VEGF-encapsulated gelatin particles (PCL/VGPs) could be used as an effective growth factor-releasing scaffold for vascular tissue engineering. This approach has potential applications in stem cell therapies and tissue regeneration by guiding cell behavior and promoting angiogenesis.

Additionally, Hariharan Ezhilarasu et al. developed functionalized core/shell nanofibers using coaxial electrospinning, incorporating PCL, GEL, SF, and VEGF at two concentrations (150 ng and 250 ng) to mimic the native extracellular matrix (ECM) for vascular tissue engineering [87]. These nanofibers supported cell adhesion, migration, and proliferation, creating a favorable environment for vascular tissue regeneration. The VEGF-functionalized scaffolds showed significantly higher surface hydrophilicity and promoted angiogenesis by inducing cell morphology changes and proliferation. The scaffolds containing SF enhanced mechanical properties and facilitated controlled VEGF release, which supported the differentiation of human mesenchymal stem cells (hMSCs) into vascular smooth muscle cells (vSMCs). Among the scaffolds, the PCL/GEL/SF/VEGF (250 ng) nanofibers demonstrated the best potential for sustained VEGF release and new tissue regeneration, making them highly effective for vascular tissue engineering and promising candidates for bypass surgery and regenerative medicine applications.

In another study, Yinxin Fu et al. explored the potential of a novel biomaterial created from human urine-derived stem cells (USCs) combined with polycaprolactone/gelatin (PCL/GEL) for wound healing applications. This engineered biomaterial was assessed for its ability to promote tissue regeneration and facilitate the healing process [88]. USCs were successfully isolated from urine samples and demonstrated differentiation potential into osteoblasts, adipocytes, and chondrocytes. The PCL/GEL composite membranes, fabricated via twin-nozzle electrospinning, exhibited suitable mechanical properties and good biocompatibility. When seeded onto PCL/GEL membranes, USCs significantly enhanced the healing of full-thickness skin wounds in rabbits, accelerating re-epithelialization, collagen formation, and angiogenesis compared to PCL/GEL alone or untreated wounds. Additionally, USC-conditioned medium promoted endothelial cell migration, proliferation, and tube formation. This study demonstrated that USCs, in combination with PCL/GEL nanofibrous membranes, offer a novel and effective therapeutic approach for skin regeneration, enhancing wound closure and angiogenesis.

Further contributions include the study performed by Lijun Zhang et al., which developed a prevascularized hMSC/ECM sheet by co-culturing human mesenchymal stem cells (hMSCs) and endothelial cells (ECs) on a naturally derived nanofibrous extracellular matrix (ECM) scaffold [89]. Initially, hMSCs were cultured under hypoxic conditions (2% O_2_) to preserve their stemness and enhance the secretion of angiogenic factors such as VEGF, basic fibroblast growth factor (bFGF), and angiopoietina-1 (Ang-1), which stabilize and support vascular network maturation. Subsequently, ECs were added to form microvessels under normoxic conditions (20% O_2_), resulting in a branched and mature vascular network. The hypoxic culture maintained hMSC multipotency and improved the microvasculature structure, with hMSCs functioning as pericytes to stabilize the vasculature. The prevascularized hMSC/ECM sheet demonstrates great potential for engineering fully biological, mechanically robust, and prevascularized 3D tissues for diverse regenerative medicine applications.

Small-diameter vascular grafts (SDVGs) often face issues of thrombosis and graft failure due to the lack of a functional endothelium. To address this, researchers designed electrospun PLLA scaffolds incorporating acetylsalicylic acid (ASA) as an anticoagulant, with their surfaces coated in amniotic membrane (AM) lysate. The scaffolds displayed the required tensile strength, excellent hemocompatibility, and a controlled release of ASA over seven days. The AM coating significantly improved cytocompatibility, facilitating endothelial cell adhesion, proliferation, and the differentiation of Wharton’s jelly-derived mesenchymal stem cells into endothelial-like cells. These findings demonstrate the potential of AM-coated ASA-PLLA scaffolds for creating biocompatible and effective SDVGs [90].

Table 5 reported examples of vascular differentiation induced on nanofibrous scaffolds specifying the type of nanofibers, the mechanical properties, the source of MSCs and the additional factors used to induce the differentiation.

## 3. Future Research Directions

The application of nanofibrous scaffolds to induce MSC differentiation for soft tissue regenerative applications holds immense potential, yet several gaps and challenges persist [92]. Until now, there has been limited understanding of the precise molecular mechanisms by which nanofibrous scaffolds influence MSC differentiation. Future studies should focus on elucidating the interplay between scaffold properties (e.g., stiffness, fiber alignment, and surface chemistry) and intracellular signaling pathways, also exploiting advanced omics techniques (transcriptomics, proteomics, and metabolomics) that can provide deeper insights into how scaffold cues drive lineage-specific differentiation [93]. Additionally, the materials used to produce the scaffolds play a crucial role in directing the differentiation of MSCs, highlighting the importance of material selection in regenerative medicine [94]. Currently, synthetic polymeric materials such as PLA, PCL, and PLGA are widely used in electrospun scaffolds for MSC differentiation in soft tissue engineering due to their biocompatibility, biodegradability, and tunable properties. PLA offers good stiffness, strength, and a suitable environment for differentiation, while PCL provides flexibility, prolonged degradation, and excellent processability. PLGA combines the benefits of both, with a tunable degradation rate and versatility that support drug delivery and angiogenesis [95]. The development of novel biomaterials, such as peptide-based nanofibers, decellularized ECM-derived fibers, and hybrid organic-inorganic scaffolds, holds promise for enhanced biocompatibility and functionality [96].

Another important aspect to take into account is the in vivo vs. in vitro correlation; many studies have been limited to in vitro settings, which fail to fully replicate the complex microenvironment of soft tissues and mimic conditions for MSC differentiation [97]. Future research should aim to bridge the gap between in vitro findings and in vivo performance by developing more physiologically relevant models. One possible strategy could be to include sufficient vascularization within scaffolds to support cell survival, integration, and differentiation [98]. Research should focus on incorporating pro-angiogenic cues, such as vascular endothelial growth factor (VEGFA) or pre-vascularized constructs, into nanofibrous scaffolds [99]. Moreover, the interaction of scaffolds with the host immune system significantly impacts their regenerative potential, influencing MSCs’ fate. Investigating scaffold designs that promote a favorable immune response while minimizing fibrosis or rejection will be crucial for clinical translation [100,101]. Finally, to assess clinical applications, long-term in vivo studies are needed to evaluate scaffold integration, biodegradation, the durability of regenerated tissue function, and the behavior of MSCs. It is also crucial to monitor the differentiation and progression of MSCs over the long term to ensure that they do not transform into tumorigenic forms [102,103].

Several patented products have been developed that focus on the use of electrospun scaffolds to facilitate the proliferation and differentiation of mesenchymal stem cells (MSCs) for tissue engineering applications (Table 6).

Few clinical trials are currently underway on the application of scaffolds and MSCs for soft tissue regeneration. One notable study performed by Peking University (NCT05789914) used an extracellular matrix scaffold of small intestinal submucosa (SIS) membrane to promote the differentiation of bone marrow mesenchymal stem cells into osteoblasts. The SIS membrane can be used in soft tissue wound repair, guided bone regeneration, site preservation, and other surgical procedures and has demonstrated good therapeutic effects [108]. In another study, an engineered synthetic scaffold (device component) seeded and cultured with the patient’s adipose-derived mesenchymal stem cells (biologic component) is designed to promote the regeneration of a structurally intact, living biological esophageal conduit (NCT05877300) [109].

The reason clinical trials involving scaffolds and MSCs are few is because there is a lack of standardization in scaffold characterization and MSC differentiation protocols. Electrospun scaffolds have shown countless advantages from both biological and functional points of view; however, many scaffold manufacturing techniques, such as electrospinning, face challenges in scalability and reproducibility [110]. Future work should explore novel fabrication methods, such as additive manufacturing or hybrid techniques, to produce clinically scalable scaffolds with precise architectural control [111]. Advances in 3D bioprinting allow for the creation of complex, patient-specific scaffold geometries. Future research could focus on integrating nanofibrous structures with 3D bioprinting to mimic tissue-specific architectures [112]. Considering the growing use of artificial intelligence (AI) and machine learning in regenerative medicine, it will be possible to optimize scaffold designs, predict MSC behavior, and analyze large datasets from scaffold–MSC interaction studies. These tools could accelerate the development of personalized regenerative therapies [113]. Research must also address the ethical considerations of MSC sourcing and the cost-effectiveness of scaffold-based therapies to ensure accessibility to the health system [114].

## 4. Conclusions

Nanofibrous scaffolds, due to their ability to mimic the ECM, have emerged as a promising tool in regenerative medicine, addressing the challenges associated with MSC differentiation and tissue repair. Various studies highlight the efficacy of different scaffold designs, including aligned and random fibers, in guiding MSC differentiation pathways. For instance, aligned fibers often outperform their random counterparts in applications such as cardiomyogenic and tendon differentiation by mimicking the anisotropic nature of native tissues. The material composition of scaffolds plays a crucial role in determining their mechanical properties and biological compatibility. Natural polymers, such as collagen and fibrin, offer excellent biocompatibility but may lack the mechanical strength required for load-bearing tissues. In contrast, synthetic polymers like polycaprolactone (PCL) and polylactic acid (PLA) provide suitable mechanical properties but may require functionalization to enhance cell interactions. Composite scaffolds combine the strengths of both natural and synthetic materials, achieving a balance between biocompatibility and mechanical performance. The findings highlight the importance of integrating mechanical and biochemical cues in scaffold design to replicate the native microenvironment of target tissues. Despite these advancements, significant challenges remain. Currently, no study has yet reached the clinical trial stage due to difficulties in guaranteeing consistent and reproducible results across studies. Furthermore, ensuring the long-term functional integration of engineered tissues with the host remains a critical step.

In conclusion, nanofibrous scaffolds with MSCs hold immense potential to revolutionize regenerative medicine. Future research should focus on addressing current limitations and facilitating the translation of these technologies into clinical settings, ensuring safe, effective, and accessible solutions for tissue repair and regeneration**.**

## Figures and Tables

**Table 1:** Cardiomyogenic differentiation induced on nanofibrous scaffolds.

Nanofibers	Mechanical Properties	MSCs Source	Supplemented Factors	Ref.
PCL-GEL	2 ± 1 MPa (random) 1 ± 0.7 MPa (aligned)	hMSCs	5-aza	[19]
PCL	-	UCB hiPSC	5-aza GSK3 inhibitor (6 μM)	[20]
PLA-PCL	6.41 ± 0.48 MPa	Ad-MSCs	5-aza and TGF-β	[21]
PCL/PANI	-	Ad-MSCs	5-aza and TGF-β	[22]
PVA-Ch-CNT	PVA-Ch = 30 ± 3.01 MPa PVA-Ch-CNT1 = 130 ± 3.605 MPa PVA-Ch-CNT3 = 121 ± 2.516 MPa PVA-Ch-CNT5 = 101 ± 1.527 MPa	Rat	20 μL 5-aza, 1.5 μL AA 1 ng·ml^−1^ TGF-β	[23]
PCL/AV/VB12/SF/AuNPs	PCL = 4.5 MPa PCL/Collagen = 1.56 MPa PCL/SF/AV = 3.27 MPa PCL/SF/AV/VB12/AuNPs = 2.56 MPa	hMSCs (PT-2501; Lonza)	-	[24]
BSA/PVA/AuNPs	4.77 MPa	hMSCs (PT-2501; Lonza)	5-aza	[25]
Collagen I	-	BM-MSCs	10 μM 5-aza	[26]
PCL/Collagen (Aligned)	3 ± 1 MPa	hMSCs (PT-2501; Lonza)	3 μM 5-aza	[27]
PCL/G-VEGF	E = 8.48 ± 0.53 MPa Tensile Strength = 3.82 ± 0.91 MPa Elongation at Break = 35.8 ± 4.8%	hMSCs (PT-2501; Lonza)	10 μM 5-aza	[28]
PGS/Fbg	3.28 ± 0.31 MPa	BM-MSCs	VEGF	[29]
Polyamide	-	Ad-MSCs	-	[30]
FN-PCL	Elongation at break = 7.9%	UCB	FN Cyclosporine	[31]
SF-PCL	120 MPa	BM-MSCs	5-aza	[32]
PAN HG yarns	100 MPa	Ad-MSCs Chicken CMs	NaOH TGF-β1	[33]
PU	-	BM-MSCs	10 µM 5-aza	[34]
Sal B/MAAP-PCL/GEL	UTS = 40 ± 2.2 MPa	H9c2	2.5 μg mL^−1^ amphotericin B	[35]

**Table 2:** Epithelial differentiation induced on nanofibrous scaffolds.

Nanofibers	Mechanical Properties	MSCs Source	Supplemented Factors	Ref.
NFM	Stress Value = 13.8 MPa (NFM) 8.8 MPa (NFM + B. vulgaris)	Ad-MSCs	B. vulgaris	[37]
Ch-PVA + Silk	UTS = ~2.4 MPa E = ~2 MPa Strain Break = ~240%	Rat BM-MSCs	2 mM CC, 5 μg/mL Insulin, 10 ng/mL EGF, 10 ng/mL KGF	[38]
CNF hydrogel	-	Rat BM-MSCs	-	[39]
PHBV	-	BM-MSCs	400 ng/mL HC, 500 ng/mL Insulin, 1 nM T3, 10 ng/mL EGF, 1 μM vD3, 50 μg/mL L-AA	[40]
EO-PCL/PEG	UTS = 5.8 MPa	Ad-MSCs	0.4 µg/mL HC, 5 µg/mL Insulin, 1 nM T3, 10 ng/mL EGF, 1 µM VD3, 50 µg/mL L-AA	[41]
PCL/Fbg	-	Ad-MSCs	1× ITS, 0.5 μg/mL HC, 10 ng/mL EGF, 10 ng/mL KGF, 50 μg/mL L-AA	[42]
Cationic GEL/HA/CS + Sericin	-	BM-MSCs	2 mM L-Glutamin	[43]
GEL/sHA HA/CS	-	BM-MSCs	GAGs	[44]
PCL/GEL—MnO_2_–NPs	UTS = 0.327 MPa (PCL/GEL) 2.525 MPa (PCL/GEL −5% MnO_2_–NPs	Ad-MSCs	-	[45]
PCL/PGS/GEL	E = 1.32 ± 0.27 MPa UTS = 1.23 ± 0.18 MPa	Ad-MSCs	50 ng/mL VEGF	[46]
Collagen/nanoHA mats	E = 8.46 ± 1.05 kPa UTS = 428 ± 54 kPa	BM-MSCs	-	[47]
PDLLA/Lam—a PDLLA/NaOH	-	MSCs	-	[48]
PVA/SS	-	A549	-	[49]

**Table 3:** Myogenic differentiation induced on nanofibrous scaffolds.

Nanofibers	Mechanical Properties	MSC Source	Supplemented Factors	Ref.
PLGA/PU PLGA/PU/polyP	UTS = 15.12 ± 2.16 MPa (PLGA/PU) 13.23 ± 1.25 MPa (PLGA/PU/polyP)	Ad-MSCs	30 mM AA, 5 ng/mL TGF-β1	[51]
PAN-PEO	E = 109.42 ± 4.55 MPa Elongation at break = 28.42 ± 2.12%	Ad-MSCs	30 mM AA, 5 ng/mL TGF-β1	[52]
PANi-PAN	-	hBM-MSCs	HS, hydrocortisone, and dexamethasone.	[53]
PVDF-TGFβ	E = 9.15 MPa Strain% = 61.7%	Ad-MSCs	2.5 ng/mL TGFβ1 30 mM Ascorbic Acid	[54]
PLGA	UTS = 11.32 ± 2.02 MPa Elongation at break = 32.43 ± 1.97%	hiPSCs	30 mM AA, 5 ng/mL TGF-β1	[55]
PCL/NP(TGF)-PLLA	-	hWJ-derived UC-MSCs	30 mM AA, 2.5 ng/mL TGF-β1	[56]
PLA-PCL/ATE/HA	E = 14.93 ± 0.13 MPa	Ad-MSCs	-	[57]
HPCL	-	Ad-MSCs	100 U/mL heparin	[58]
PCL/Collagen I	-	Lewis 1WR2 Rat (BM-MSC) + Mb	0.4 μg/mL DXM, 1 ng/mL bFGF, HGF (10, 30, 60, 100 ng/mL), IGF-1 (5, 10, 30, 60 ng/mL), 10 ng/mL HGF + 10 ng/mL IGF1	[59]
PCL/Collagen I	-	Lewis Rat (BM-MSC, Ad-MSC) + Mb	AIM V, AIM V + 0,1% Ultroser^®^ G, DMEM/Ham’s F12 + 0,2% Ultroser^®^ G	[60]
PCL/Collagen I	-	Ad-MSC + Mb + SCs	2% DHS, 1% L-GlutaMAX, 0.4 μg/mL DXM, 1 ng/mL bFGF	[61]
SF/Fe	E = 233.62 ± 5.27 MPa Elongation at break = 10%	MSCs	-	[62]
PαAPz-A	-	iPSCs BM-MSCs	82.5 μg/mL L-AA 2 ng/mL TGF-β1	[63]

**Table 4:** Tendon differentiation induced on nanofibrous scaffolds.

Nanofibers	Mechanical Properties	MSCs Source	Supplemented Factors	Ref.
GEL/PCL PGA	-	Ad-MSCs	-	[65]
PCL/PTMC-MA (1:3)	E = 31.13 ± 1.30 MPa Maximum Stress at Break = 23.80 ± 3.44 MPa Yield Strain = 170 ± 22%	Mouse C57BL/6	-	[66]
Aligned PCL/Collagen multiscale fibers	-	hMSCs (Lonza)	-	[67]
PCL Braided	Ultimate elongation (mm) = 24.47 ± 5.61 Stiffness (N/mm) = 11.25 ± 5.04	BM-MSCs	50 μg/mL ascorbic acid 2-phosphate + 10 ng/mL TGF-β3	[68]
PLLA Braided	Ultimate elongation (mm) = 17.01 ± 3.86 Stiffness (N/mm) = 5.94 ± 2.68			
PCL Stacked	Ultimate elongation (mm) = 9.47 ± 1.15 Stiffness (N/mm) = 36.51 ± 7.33			
PLLA Stacked	Ultimate elongation (mm) = 6.43 ± 3.06 Stiffness (N/mm) = 24.31 ± 7.61			
PCL/PLA	-	Ad-MSCs	20 ng/mL TGF-β3 hTC/hUVEC	[69]
PLGA/PLA HY	-	Ad-MSCs	20 ng/mL TGF-β3, TGF-β4	[70]
PPDO/SF	UTS = 31 ± 1 MPa PPDO WNS 25 ± 1 MPa PPDO WNS 4/1 17 ± 1 MPa PPDO WNS 2/1	Ad-MSCs	20 ng/mL TGF-β3	[71]
SF/P3HB	UTS = 197.0 ± 7.7 MPa	Ad-MSCs	GDF-5	[72]
PCL dissolved in HFIP	UTS = ~55 MPa E = ~100 MPa	BM-MSCs	1% P/S	[73]
PCL	Young’s modulus = 121.5 ± 3.8 MPa Yield stress = 6.3 ± 0.09 MPa Yield Strain = ~10%	BM-MSCs	50 μg/mL L-AA 2-phosphate, 0.25 μg/mL amphotericin B, 100 ng/mL CTGF	[74]
GO-PLGA	UTS = 2.37 ± 0.31 MPa (PLGA) 2.05 ± 0.29 MPa (GO-PLGA)	BM-MSCs	-	[75]
Ch-PLLA-GEL-PEO	UTS = 14.23 ± 1.08 MPa (aligned), 2.43 ± 0.28 MPa (randomly) E = 325.01 ± 25.05 MPa (aligned), 74.46 ± 12.99 MPa (randomly)	hiPSC	2 mM L-glutamine, 10 ng/mL bFGF, 1% AA,	[76]
Aligned and Randomly oriented PLLA	E = 24.42 ± 2.20 MPa (aligned) 20.86 ± 3.56 MPa (randomly)	C3H10T1/2 Rat MSCs	10 mM β-glycerophosphate, 0.1 mM DXM, 50 mg/mL AA	[77]
PCL B@P S + B@P	-	SD Rats BM-MSCs	SDF-1α, BMP-2	[78]
PS	-	BM-MSCs	-	[79]
SF/GelMa	UTS = 500–1000 kPa (%GElMa dependent)	BM-MSCs	-	[80]

**Table 5:** Vascular differentiation induced on nanofibrous scaffolds.

Nanofibers	Mechanical Properties	MSC Source	Supplemented Factors	Ref.
Pectin HG	-	BM-MSCs	10 ng/mL TGFβ1, 50 ng/mL VEGF	[82]
PCL (SES diameter 1,4 ± 0,2 μm MEW diameter 15,2 ± 4,8 μm)	-	BM-MSCs	30 μM ASAP, 5 ng/mL TGF-β1	[83]
PCL/GEL co-spun functionalized with Hep-PG	Strain = 50%	Ad of Sprague–Dawley (SD) Rats	VEGF	[84]
PLCL + S-PLCL + H-PLCL	UTS = 11.38 ± 0.34 MPa E = 0.99 ± 0.10 MPa Elongation = 776.4 ± 44.9%	hMSCs	-	[85]
PCL	-	-	VEGF	[86]
Core/shell (PCL + GEL 3:6 150 ng SF + 250 ng VEGF)	E = 3.0 MPa (PCL/GEL) 3.3 MPa (PCL/GEL + SF) 2.8 MPa for 150 ng, 2.6 MPa for 250 ng (PCL/GEL + SF + VEGF)	hMSCs from Lonza, Singapore	VEGF	[87]
PCL/GEL	E = 0.86 MPa	USCs	-	[88]
ECM derived from decellularized hDF cell sheets	-	hMSCs	-	[89]
PLLA	-	hWJ from the UC	ASA as an antithrombogenic agent	[90]
PEA	-	10T1/2	TGF-b1	[91]

**Table 6:** Patented products developed focusing on the use of electrospun scaffolds to facilitate the proliferation and differentiation of mesenchymal stem cells (MSCs) for tissue engineering applications.

N°	Title	Field of the Invention	Ref.
US10052412B2	Electrospun electroactive polymers for regenerative medicine applications	Synthetic electroactive, or piezoelectric, biomaterial useful as an electroactive scaffold for repairing tissues.	[104]
US20190030211A1	Hydrogel scaffold for three-dimensional cell culture	A scaffolding system that includes a thermosensitive hydrogel and a biodegradable polymer blended into a composite, electrospun microfibrous structure.	[105]
US20110293685A1	Scaffolds for tissue engineering and regenerative medicine	The present invention relates to the field of tissue regeneration and replacement.	[106]
EP4374006A1	Method for the production of a shape-memory tissue and relative uses	The present invention involves a method for producing a shape-memory tissue using polymeric matrices that can temporarily alter their shape in response to an external stimulus. These matrices are also designed to support the delivery of cells and/or drugs.	[107]

## Abbreviations

10T1/2	Mouse embryonic multipotent mesenchymal progenitor cell line
5-Aza	5-Azacytidine
A549	Human lung epithelial cells
AA	Ascorbic acid
ACL	Anterior cruciate ligament
ACTA1	Skeletal alpha actin 1
Ad	Adipose tissue-derived
ADH	Alcohol dehydrogenase
ADM	Acellular dermal matrix
Ad-MSCs	Adipose-derived mesenchymal stem cells
AM	Amniotic membrane
Ang-1	Angiopoietina-1
ASA	Acetylsalicylic acid
ASAP	L-ascorbic acid 2-phosphate
ASMA	Alpha-smooth muscle actin
ATE	Adipose tissue extract
AuNPs	Gold nanoparticles
AV	Aloe vera
B. vulgaris	Beta vulgaris
B@P	BMP-2-loaded CS/HA multilayer-modified PCL scaffold
bFGF	Basic fibroblast growth factor
BM	Bone marrow-derived
BMP-2	Bone morphogenetic protein-2
BM-MSCs	Bone marrow mesenchymal stem cells
BNFSs	Braided nanofibrous scaffolds
BSA	Bovine serum albumin
CC	Calcium chloride
CK-19	Cytokeratin-19
CK-MB	Creatine kinase-MB
CMs	Cardiomyocytes
CNF	Chitin nanofiber
CNT	Carbon nanotube
Ch	Chitosan
CS	Chondroitin sulfate
CTGF	Connective tissue growth factor
cTnI	Troponin I
cTnT	Troponin T
Cx43	Connexin 43
DCM	Dichloromethane
DHS	Donor horse serum
DM	Differentiation medium
DMF	Dimethylformamide
DXM	Dexamethasone
E	Young’s modulus
EBs	Embryonic bodies
ECG	Electrocardiogram
ECM	Extracellular matrix
ECs	Endotelial cells
EGF	Epidermal growth factor
EO	Emu oil
Fb	Fibrin
Fbg	Fibrinogen
FBS-HI	Fetal bovine serum—heat-inactivated
FE	Field emission
FGF-2	Fibroblast growth factor-2
FN	Fibronectin
GA	Glutaraldehyde
GEL	Gelatin
GELMa	Gelatin methacryloyl
GDF-5	Growth and differentiation factor-5
GO	Graphene oxide
H9c2	Rat cardio myoblast
Hb	Hemoglobin
HA	Native hyaluronan
HaCaT	Keratinocyte cell line from adult human skin
HC	Hydrocortisone
HFIP	1,1,1,3,3,3-Hexafluoroisopropanol
HG	HydroGel
HGF	Hepatocyte growth factor
hiPSCs	Induced pluripotent stem cells
hMSCs	Human mesenchymal stem cells
HPCL	Heparin polycaprolactone
hTCs	Human tenocytes
hUVECs	Human umbilical vein endotelial cells
hWJ	Human Wharton’s jelly-derived
HY	Hybrid yarns
ICC	Immunocytochemistry
IGF-1	Insulin-like growth factor 1
IMDM	Iscove’s modified Dulbecco’s medium
ITS	Insulin–transferrin–selenium
IVL	Involucrin
KGF	Keratinocyte growth factor
L-AA	L-ascorbic acid
LbL	Layer-by-layer
LDH	Lactate dehydrogenase
MAAB	Magnesium l-ascorbic acid 2 phosphate, salts
Mb	Myoblasts
MHC	Myosin heavy chain
MI	Myocardial infarction
MnO2–NPs	Manganese nanoparticles
MSC-FBS	MSC-fetal bovine serum
MSCs	Mesenchimal stem cells
MTS assay	3-(4,5-Dimethylthiazol-2-yl)-5-(3-carboxymethoxyphenyl)-2-(4-sulfophenyl)-2H-tetrazolium
MY	Microfiber yarns
MYH2	Myosin heavy chain 2
MYOG	Myogenin
N NFS	Non-composite nylon66 nanofibrous scaffold
nanoHA	Nanophased hydroxyapatite
N-B.v NFS	Composite nylon66 nanofibrous scaffold
NGF	Nerve growth factor
NP(TGF)	TGF-β1-loaded chitosan nanoparticles
P/S	Penicillin/streptomycin
PAN	Polyacrylonitrile
PαAPz-A	Poly(organophosphazene)—L-alanine
PEO	Polyethylene oxide
PCL	Polycaprolactone
PDGF	Platelet-derived growth factor
PEG	Polyethylene glycol
PenStrep	Penicillin-streptomycin
PG	Propylene glycol
PGS	Poly (glycerol sebacate)
PHBV	Poly 3-hydroxybutyrate-co-3-hydroxyvalerate
PLA BNFSs	Poly(lactic acid) braided nanofibrous scaffolds
PLCL	Poly(L-lactide-co-ε-caprolactone)
PLGA	Poly(lactic-co-glycolic acid)
PLLA	Poly-L-lactic acid
PDLLA/Lam—a PDLLA/NaOH	Hydrolyzed poly-L-lactic acid with laminin
PEA	Poly(ester amide)
poly-P	Poly-phosphate
PPDO	Poly(p-dioxanone)
PS	Polystyrene
PVA	Polyvinyl alcohol
PU	Polyurethane
PTMC-MA	Poly(trimethylene carbonate)-methacrylated
RT-PCR	Real-time PCR
S + B@P	SDF-1α- and BMP-2-loaded CS/HA multilayer-modified PCL scaffold
Sal B	Salvianolic acid B
SCs	Schwann cells
SDVGs	Small-diameter vascular grafts
SEM	Scanning electron microscopy
SF	Silk fibroin
SF/Fe	Silk fibroin/Fe3O4 nanoparticles
sGAG	Sulfated glycosaminoglycans
sHA	Sulfated hyaluronan
SJES	Stable jet electrospinning
SM-22a	Smooth muscle 22 alpha
SMC	Smooth muscle cells
SP	Substance P
SS	Silk sericin
Tβ4	Thymosin β4
T3	3,3′,5-Triiodo-L-thyronine
TCPS	Tissue culture polystyrene
TE	Tissue engineering
TGF-β	Transforming growth factor-β
THF	Tetrahydrofuran
T/L	Tendon and ligament
UC	Umbilical cord
UCB	Umbilical cord blood
USCs	Urine-derived stem cells
VB12	Vitamin B12
VD3	Vitamin D3
VEGF	Vascular endothelial growth factor
VGPs	VEGF-encapsulated gelatin particles
Vim	Vimentin
vSMCs	Vascular smooth muscle cells
YM	Young’s modulus
WNSs	Wavy nanofibrous scaffolds
